# Cathepsin B in Antigen-Presenting Cells Controls Mediators of the Th1 Immune Response during *Leishmania major* Infection

**DOI:** 10.1371/journal.pntd.0003194

**Published:** 2014-09-25

**Authors:** Iris J. Gonzalez-Leal, Bianca Röger, Angela Schwarz, Tanja Schirmeister, Thomas Reinheckel, Manfred B. Lutz, Heidrun Moll

**Affiliations:** 1 Institute for Molecular Infection Biology, University of Würzburg, Würzburg, Germany; 2 University of Mainz, Institute for Pharmacy and Biochemistry, Mainz, Germany; 3 University of Freiburg, Institute of Molecular Medicine and Cell Research, Freiburg, Germany; 4 Institute of Virology and Immunobiology, University of Würzburg, Würzburg, Germany; University of Texas Medical Branch, United States of America

## Abstract

Resistance and susceptibility to *Leishmania major* infection in the murine model is determined by the capacity of the host to mount either a protective Th1 response or a Th2 response associated with disease progression. Previous reports involving the use of cysteine cathepsin inhibitors indicated that cathepsins B (Ctsb) and L (Ctsl) play important roles in Th1/Th2 polarization during *L. major* infection in both susceptible and resistant mouse strains. Although it was hypothesized that these effects are a consequence of differential patterns of antigen processing, the mechanisms underlying these differences were not further investigated. Given the pivotal roles that dendritic cells and macrophages play during *Leishmania* infection, we generated bone-marrow derived dendritic cells (BMDC) and macrophages (BMM) from *Ctsb* ^−/−^ and *Ctsl* ^−/−^ mice, and studied the effects of Ctsb and Ctsl deficiency on the survival of *L. major* in infected cells. Furthermore, the signals used by dendritic cells to instruct Th cell polarization were addressed: the expression of MHC class II and co-stimulatory molecules, and cytokine production. We found that *Ctsb* ^−/−^ BMDC express higher levels of MHC class II molecules than wild-type (WT) and *Ctsl* ^−/−^ BMDC, while there were no significant differences in the expression of co-stimulatory molecules between cathepsin-deficient and WT cells. Moreover, both BMDC and BMM from *Ctsb* ^−/−^ mice significantly up-regulated the levels of interleukin 12 (IL-12) expression, a key Th1-inducing cytokine. These findings indicate that *Ctsb* ^−/−^ BMDC display more pro-Th1 properties than their WT and *Ctsl* ^−/−^ counterparts, and therefore suggest that Ctsb down-regulates the Th1 response to *L. major*. Moreover, they propose a novel role for Ctsb as a regulator of cytokine expression.

## Author Summary

The emergence of resistance to the available drugs against cutaneous leishmaniasis emphasizes the need of new chemotherapeutic approaches. Cysteine proteases from *Leishmania* are important virulence factors and, therefore, interesting drug targets. Studies on inhibitors against these enzymes during *Leishmania major* infection in mice had shown that host equivalents of these proteases are also affected, namely cathepsin B and cathepsin L. The inhibition of cathepsin B resulted in immune-mediated protection, while inhibition of cathepsin L caused susceptibility to the parasite. In the present study, we investigated the effect of cathepsin deficiency on the signals used by dendritic cells to orchestrate the T helper (Th)-mediated immune response against *L. major* and the control of parasite proliferation within infected macrophages. The results demonstrate that cathepsin B-deficient dendritic cells express higher levels of the antigen-presenting MHC class II molecules than WT and cathepsin L-deficient cells. Surprisingly, dendritic cells and macrophages deficient for cathepsin B showed higher expression of the protective Th1-inducing cytokine IL-12. Therefore, we propose a novel role of this protease as a regulator of cytokine expression. Altogether, these findings suggest that cathepsin B down-regulates the Th1 response to *L. major*, and, in its absence, antigen-presenting cells express signals protecting against the parasite.

## Introduction

Worldwide, 2 million new cases of leishmaniasis occur every year. It is endemic in 98 countries, where 350 million people are considered to be at risk, largely affecting “the poorest of the poor” [1], [2]. The cutaneous form of leishmaniasis is characterized by lesions that heal over the course of months or years, and leave permanent scars that can be disfiguring or disabling [1], [2]. Control of *Leishmania* within the host is mediated by innate and adaptive immune responses. Experimental mouse models of *Leishmania major* infection first documented the relevance of Th1/Th2 polarization for resistance and susceptibility to the disease *in vivo* [3]. In resistant mouse strains, such as C57BL/6, a contained and self-healing development of the disease appears, mediated by a protective Th1 immune response. Th1 cells secrete IFN-γ, which induces the nitric oxide (NO)-mediated killing of the amastigote form of the parasite within phagosomes in macrophages. In contrast, infection of BALB/c mice with *L. major* causes a non-healing Th2 form of the disease, characterized by expression of the cytokines IL-4, IL-13, and IL-10.

The key role of dendritic cells (DC) in inducing cell-mediated immune responses against leishmaniasis has been extensively documented [4], based on their capacity to migrate to draining lymph nodes after capture of *Leishmania* parasites and to induce Th cell polarization. Several subsets of DC have been reported to perform this function, including Langerhans cells [5], dermal DC [6], lymph node resident DC [7], and monocyte-derived DC [8], [9]. In order to instruct Th cell polarization, DC use three main signals: (1) antigen presentation via MHC class II molecules, (2) the expression of co-stimulatory molecules and (3) cytokine secretion. Quantitative and qualitative differences in these signals are crucial for Th cell polarization [10]. Among these signals, IL-12 is a key cytokine for the development of a protective Th1 immune response. Neutralization of IL-12 by antibodies leads to susceptibility to *Leishmania* infection in otherwise resistant mice [11], [12]. Conversely, treatment of BALB/c mice with IL-12 resulted in a protective Th1 response [13]. DC have been reported to be the primary source of IL-12 in lymphoid tissues [14], with variations depending on the DC subset, maturation status, and whether promastigotes or amastigotes are used [15]. Macrophages, on the other hand, are considered as main host cells for *Leishmania* parasites, where freshly inoculated promastigotes find a niche for differentiating into amastigotes and proliferating. Macrophages are not able at all to produce IL-12 in response to *L. major* [16], [17], even if they are further stimulated with lipopolysaccharide (LPS) [18], [19], reflecting the extent of the silencing that *L. major* induces in its host.

Silencing of infected cells has been attributed to different virulence factors. Some of them are cysteine proteases [20], which impair NF-κB signaling in macrophages [21] and are also important for autophagic and differentiation processes in the parasite [22]. Therefore, they are interesting targets for drug development [23], [24]. However, they also have homologs in mammals. Few studies have addressed the effects that unspecific inhibition of host cathepsins would have on the immune response against *L. major*. Maekawa et al. reported that treatment of *L. major*-infected BALB/c mice with the Ctsb inhibitor CA074 triggered a protective Th1 immune response [25], [26]. Treatment of these mice with the Ctsl inhibitor CLIK148, on the other hand, caused a stronger Th2 response [27], even in resistant mouse strains [28]. The authors showed that the inhibitors had no direct effect on the proliferation of the parasite but that the host cell cathepsins were inhibited, and hypothesized that lack of Ctsb or Ctsl would lead to different patterns for proteolytic processing of *L. major* antigens. It had remained unclear, however, how the inhibition of Ctsb and Ctsl activity could have such effects in Th polarization. Thus, further investigation is needed to understand the involvement of Ctsb and Ctsl in the immune response during leishmaniasis.

In the present study, we used cathepsin B (*Ctsb^−/−^*)- and cathepsin L (*Ctsl^−/−^*)-deficient bone marrow-derived DC (BMDC) and bone marrow-derived macrophages (BMM) to determine the role of these proteases for the signals that DC use to instruct Th cell polarization in response to *L. major* promastigotes: the expression of MHC class II and co-stimulatory molecules, and the production of cytokines. Furthermore, we studied the effect of these cathepsins on parasite proliferation in infected cells. Our results indicate that in this model of infection, cathepsin B plays a significant role not only in the expression of antigen-presenting MHC class II molecules, but also in the regulation of IL-12 production.

## Methods

### Culture media

Complete RPMI medium was prepared by supplementing RPMI 1640 medium (with phenol red or phenol red-free, as indicated; Invitrogen, Darmstadt, Germany) with heat-inactivated fetal calf serum (FCS, 10% v/v; PAA Laboratories, Pasching, Austria), L-glutamine (final concentration 2 mM; Biochrom AG, Berlin, Germany), HEPES (pH 7.2, 0.01 M; Invitrogen, Darmstadt, Germany), penicillin G (0.2 U/ml; Sigma-Aldrich, Taufkirchen, Germany), gentamicin (0.05 mg/ml; Sigma-Aldrich), and 2-mercaptoethanol (0.05 mM; Sigma-Aldrich). In addition, for generation of BMM, a conditioned medium was used containing Dulbecco's Modified Eagles Medium (DMEM; Invitrogen), heat-inactivated FCS (10% v/v; PAA Laboratories), heat-inactivated horse serum (0.5%; Invitrogen), 2-mercaptoethanol (0.05 mM; Sigma-Aldrich), nonessential amino acids (Invitrogen), HEPES (0.01 M; Invitrogen), L-glutamine (4 mM; Biochrom) and L929 supernatant (15% v/v). *L. major* promastigotes were cultured in a biphasic medium consisting of a solid base of rabbit-blood agar (Elocin-lab, Gladbeck, Germany) plus a liquid phase of RPMI medium without phenol red.

### Preparation of BMDC and BMM

BMDC and BMM were generated from bone marrow progenitors as described previously [23], [29], [30] from female BALB/c, C57BL/6, C57BL/6 *Ctsb^−/−^* and C57BL/6 *Ctsl^−/−^* mice (6–12 weeks old). The generation of *Ctsb^−/−^* and *Ctsl^−/−^* mice has been described previously [31]–[33]. Briefly, total bone marrow cells were flushed from femurs and tibiae. To generate DC, the cell number was determined by trypan blue staining, and 0.2×10^6^ cells/ml bone marrow cells were cultured in complete RPMI 1640 medium in the presence of recombinant murine granulocyte-macrophage colony-stimulating factor (GM-CSF, 0.04 µg/ml; Invitrogen) at 37°C, 5% CO_2_. Cultures were fed with complete RPMI medium supplemented with GM-CSF on days 3 and 6. At day 8, the non-adherent cells were collected, washed with complete RPMI medium and resuspended at 2×10^6^ cells/ml in complete RPMI medium. BMM were generated by culturing 0.67×10^6^ cells/ml of total bone marrow progenitors as described above in conditioned DMEM at 37°C and 5% CO_2_. On day 6, the culture medium was removed carefully and replaced with cold RPMI complete medium, and the petri dishes were kept on ice for 10 min. Thereafter, the macrophages were removed with a cell scrapper, washed with fresh complete medium without phenol red, and resuspended at 2×10^6^ cells/ml.

As quality control, the morphology of the obtained BMDC and BMM was analyzed. Part of the cells was used for cytospin preparations stained with Diff-Quik II dye (Medion Diagnostics, Düdingen, Switzerland) according to the manufacturer's instructions, and observed under the light microscope. Furthermore, the expression of the phenotypic markers CD11c in DC and F4/80 in macrophages was assessed by flow cytometry as described below.

### Transmission electron microscopy (TEM)

The morphology of BMDC and BMM from WT, *Ctsb^−/−^* and *Ctsl^−/−^* mice was additionally analyzed by TEM. Samples of the obtained cells were prepared for TEM using OsO_4_ and uranyl acetate as contrasting agents, following the protocol previously described by Schurigt et al. [24].

### Parasites

The *L. major* isolate MHOM/IL/81/FE/BNI was maintained by continuous passage in BALB/c mice, and promastigotes were grown *in vitro* in blood-agar cultures as described previously [34] at 27°C, 5% CO_2_ and 95% humidity. In order to preserve maximal infectivity, only promastigotes passaged 5 to 8 times were used for *in vitro* infection experiments. In addition, two different transgenic *L. major* strains were used in some experiments: a luciferase-transgenic strain (Luc-tg) previously described [30], and an eGFP-transgenic strain (eGFP-tg). For the preparation of *L. major* antigen (LmAg), stationary-phase WT promastigotes were washed three times in phosphate-buffered saline (PBS), resuspended at 1×10^9^/ml in PBS, and subjected to three cycles of freezing in liquid nitrogen and thawing. The aliquots were stored at −80°C and each aliquot was thawed not more than twice. Heat-killed parasites (HK) were prepared by incubating a parasite suspension of 1×10^9^/ml in RPMI medium for 30 min at 80°C.

### Generation of the eGFP-tg *L. major* strain

The enhanced-green fluorescent protein (eGFP)-coding region was cut from pEGFP-N1 (Clontech, Saint-Germain-en-Laye, France) by BamHI-NotI (Promega, Mannheim, Germany) and cloned into the Bglll-NotI-restricted *Leishmania* expression vector pLEXSY-hyg2 (Jena Bioscience, Jena, Germany), which contains a marker gene for selection with hygromycin. The generated plasmids were linearized by SwaI (New England Biolabs, Frankfurt, Germany), and the parasites were transfected by electroporation. eGFP and HYG were integrated into the 18S rRNA locus of *L. major* by homologous recombination. For *in vitro* experiments, promastigotes were grown in blood-agar cultures supplemented with 50 µg/ml hygromycin under the same conditions as WT and Luc-tg *L. major* promastigotes. In order to maintain their virulence, eGFP-tg *L. major* parasites were passaged in female BALB/c mice. The stability of the integrated eGFP without further selection by hygromycin was assessed *in vitro* and *in vivo* by flow cytometry.

### Measurement of intracellular Luc-tg *L. major* amastigotes

Intracellular amastigotes in BMM from *Ctsb^−/−^* and *Ctsl^−/−^* mice, and their WT C57BL/6 counterparts, were measured with the method described by Bringmann et al. [30]. Briefly, 200 µl of a 2×10^5^ cells/ml suspension of BMM in phenol red-free complete medium were seeded into 96-well plates with clear bottoms (Greiner Bio-One, Frickenhausen, Germany) and were incubated for 4 hours to allow cell adhesion. The medium was then removed, and 200 µl of a 3×10^6^ cells/ml suspension of Luc-tg *L. major* promastigotes were added at an infection ratio of 1∶15 and incubated for 24 hours at 37°C, 5% CO_2_. Any remaining extracellular parasites were eliminated by washing 3 times with medium, and 200 µl of the phenol red medium were added. After further 24 hours incubation at 37°C, 5% CO_2_, 50 µl of the luciferin-containing lysis buffer Britelite Plus (PerkinElmer, Waltham, USA) were added to each well. The plate was incubated in the dark for 5 min at room temperature (RT), and the resulting luminescence was measured as counts per second (CPS), with a Victor×Light 2030 luminometer (PerkinElmer).

### Intracellular parasite count by fluorescence microscopy

5×10^5^ BMM from WT, *Ctsb^−/−^* and *Ctsl^−/−^* mice were seeded in duplicates into chambered cover glasses, in a final volume of 250 µl of complete medium without phenol red, and incubated at 37°C, 5% CO_2_ for 4 hours to promote cell adhesion. The culture medium was removed, replaced by an equivalent volume of eGFP-tg *L. major* promastigotes at an infection ratio of 1∶15, and the cells were further incubated for 24 hours at 37°C, 5% CO_2_. The cells were then washed 3 times with warm PBS; part of the cells were incubated with Hoechst solution 0.5% v/v (Immunochemistry Technologies, Bloomington, USA) for 15 min at 37°C protected from the light, followed by washing 3 times with warm PBS and addition of 250 µl of complete medium. Then, they were observed under a fluorescence microscope (Leica Microsystems). The rest of the cells were incubated in fresh medium for further 24 hours, stained and observed under the fluorescence microscope as described above. The amount of cells and *L. major* bodies were quantified with the Cell Counter plug-in from the ImageJ software [35], and the average parasite count per infected cell was calculated as a geometric mean (G) using the formula 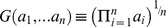, where 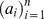 represents the sequence of parasites counted for every infected cell.

### Uptake and processing of *L. major* promastigotes by BMDC

1×10^6^ BMDC/ml were harvested at day 7 of culture, plated in 6-well plates and incubated overnight at 37°C, 5% CO_2_. For some experiments, BALB/c BMDC were pre-incubated with 10 µM CA074Me (Bachem, Bubendorf, Switzerland), 10 µM CLIK148 (kindly provided by Prof. Tanja Schirmeister), or 10 µM of Z-Arg-Leu-Arg-α-aza-glycyl-Ile-Val-OMe (ZRLR, kindly provided by Dr. Timo Burster, University of Ulm, and Dr. Ewa Wieczerzak, University of Gdansk) for 4 hours prior to infection. eGFP-*L. major* promastigotes were harvested, washed 3 times in warm PBS, added to the BMDC at a 1∶5 infection ratio, and further incubated at 37°C. After 2 hours of exposure of the BMDC to the parasites, the cells were washed with warm PBS and resuspended in fresh medium at a concentration of 1×10^6^ cells/ml. Part of the cells was fixed in paraformaldehyde (PFA, 4%; Applichem, Darmstadt, Germany). The remaining cells were incubated for a total of 4 hours or 24 hours post infection, fixed, and the amount of infected cells at the different time points was determined by flow cytometry, together with the expression of maturation markers as described next.

### Flow cytometry for detection of cell surface markers

BMDC infected with e-GFP *L. major* or stimulated with LmAg (30 µl LmAg/ml, equivalent to 30 parasites per BMDC) were fixed with 4% PFA and resuspended in FACS buffer containing the following antibodies (Ab): phycoerythrin-cyanine 7 (PECy7)–conjugated anti-CD11c (BD Biosciences, Heidelberg, Germany), phycoerythrin (PE)-conjugated anti-CD86 (BD Biosciences), allophycocyanin (APC)-conjugated anti-MHC class II (Miltenyi, Bergisch Gladbach, Germany). For some assays, BMDC were infected with WT *L. major* promastigotes instead, and fluorescein isothiocyanate (FITC)-conjugated anti-CD40 (Biolegend, San Diego, USA) and FITC-conjugated anti-CD80 (eBioscience, San Diego, USA) Ab were used. Data was obtained using the MACSQuant flow cytometer (Miltenyi) and analyzed using FlowJo (Tree Star Inc., CA, USA). The expression of F4/80 in BMM was determined using FITC-conjugated anti-F4/80 Ab (Biolegend).

### Measurement of intracellular cytokines by flow cytometry

The expression of intracellular IL-12 was analyzed in BMDC after 24 hours of stimulation with LPS (1 µg/ml) at 37°C, 5% CO_2_, in the presence or absence of 10 µM CA074Me or 10 µM ZRLR, and brefeldin A (3 µg/ml, eBioscience). The cells were then incubated for 20 min in 4% PFA fixation buffer, permeabilized for 20 min at 4°C using 0.1% saponin, 1% FCS permeabilization buffer, and incubated for 1 hour with (PECy7)-conjugated anti-CD11c and PE-conjugated anti-IL-12(p40/p70, BD Biosciences. Data was obtained using the MACSQuant flow cytometer. Furthermore, cells from polarization assays described below were fixed with 2% formaldehyde for 20 min at 4°C, permeabilized for 20 min at 4°C, and stained with the following Ab diluted in permeabilization buffer: Pacific Blue-conjugated anti-CD4 (Biolegend), FITC-conjugated anti-IFN-γ (BD Biosciences), PE-conjugated anti-IL-4 (BD Biosciences), and allophycocyani-conjugated anti-IL10 (Biolegend). Data was obtained using a LSR-II flow cytometer (BD Biosciences, San Jose, USA). All results were analyzed using the software FlowJo.

### Measurement of NO production

BMM from WT and cathepsin-deficient mice were seeded and infected as described for the proliferation assay. After 24 hours of incubation, the cells were washed with phenol-free complete RPMI medium to eliminate any extracellular parasites and incubated for further 48 hours in the absence or presence of 1 µg/ml LPS. The supernatants were collected, and the concentration of nitrite (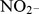) was determined by addition of 100 µl of culture supernatant to 100 µl of Griess reagent (Sigma-Aldrich) and incubation for 15 min at RT. The resulting absorbance at 540 nm was measured with the Multiskan Ascent ELISA reader (Thermo Electronic Corporation). The nitrite concentrations were determined using sodium nitrite (NaNO_3_) as a standard, and reflect the NO levels released by macrophages.

### Analysis of cytokine production

1×10^6^ BMDC were seeded in a final volume of 1 ml in 24-well plates, and were stimulated with 5×10^6^ *L. major* WT promastigotes (infection ratio 1∶5), LmAg (30 µl/ml), LPS (1 µg/ml; Sigma-Aldrich), or CpG ODN 1668 (5′-TCCATGACGTTCCTGATGCT-3′, Qiagen Operon, Cologne, Germany). The cells were further incubated for 24 or 48 hours, and the supernatants were collected. The concentration of the cytokines in the supernatants was determined by sandwich ELISA, using capture-detection Ab pairs purchased from BD Biosciences for IL-12p40, IL-6 and tumor necrosis factor alpha (TNF-α), and R&D Systems for IL-10 (Wiesbaden, Germany) following the suppliers' instructions. In addition, IL-12p70 was measured by using the IL-12p70 ELISA Ready-SET-Go kit from eBioscience according to the manufacturer's instructions. To analyze the cytokine production in BMM, 1×10^6^ cells were seeded in 500 µl into 24-well plates, together with 15×10^6^ *L. major* WT promastigotes (infection ratio 1∶15), in the presence or absence of LPS (1 µg/ml). The cells were incubated for 24 and 48 hours, and the supernatants were collected. Cytokine measurements by ELISA were performed as described above.

### Real-time PCR

Total RNA from 2×10^6^ BMDC or BMM, stimulated as described above, was isolated using the RNeasy kit (Qiagen, Hilden, Germany) according to the manufacturer's instructions. cDNA synthesis was performed using the iScript cDNA synthesis kit (BioRad, Munich, Germany) and the resulting cDNA was used at a 1∶8 dilution to assess the expression of IL-12a(p35) by real-time PCR. The real-time PCR was performed in a final volume of 25 µl per well using Maxima SYBR Green/Fluorescein qPCR Master Mix (Thermo Scientific, Schwerte, Germany) and run with a CFX96 Touch real-time PCR detection system (BioRad) for 40 cycles. The primer pairs used were: Il12p35 forward: TGGCTACTAGAGAGACTTCTTCCACAA, Il12p35 reverse: GCACAGGGTCATCATCAAAGAC; Il12p40 forward: CGTGCTCATGGCTGGTGCAAA, Il12p40 reverse: ACGCCATTCCACATGTCACTGCC. The housekeeping gene β-actin was used for normalization of the samples: β-actin forward: CATTGCTGACAGGATGCAGA, β-actin reverse: TTGCTGATCCACATCTGCTG. Non-treated (NT) BMM were used as negative control, and LPS-stimulation (1 µg/ml) was used as positive control. Relative gene expression values were calculated with the 2^− ΔΔC^_T_ method [36] using WT NT BMM at t = 6 hours as a reference.

### Th1 polarization assays

Lymph nodes and spleens were removed from OVA-specific T-cell receptor (TCR)-transgenic OT-II mice, and kept in ice-cold complete RPMI medium in 60×15 mm petri dishes. Lymphocytes and splenocytes were isolated by mechanical dissociation using the sterile plunger of a 5-ml syringe and a cell strainer (70 µm, BD Falcon, Durham, USA). Red blood cells from spleen suspensions were eliminated with ammonium chloride lysis buffer for 5 min at 37°C. Naïve CD4^+^ T cells were isolated by negative selection using the CD4^+^ T-cell enrichment kit (StemCell Technologies, Grenoble, France) following the manufacturer's instructions. The enriched cells (1×10^4^) were co-cultured with day 8 BMDC (5×10^4^) from WT C57BL/6, Ctsb^−/−^ or BALB/c mice, together with 1 mg/ml ovalbumine (OVA, Hyglos, Bernried, Germany) or 100 ng/ml OVA-peptide _327–339_ (Activotec, Cambridge, UK.), and LPS (0.1 µg/ml), in U-bottom 96-well plates, with a final volume of 200 µl/well at 37°C, 5% CO_2_. After 5 days of culture, the cells were harvested, counted, and adjusted to a concentration of 1×10^6^ cells/ml for re-stimulation with phorbol 12-myristate 13-acetate (PMA,10 ng/ml, Sigma-Aldrich), ionomycin (1 µg/ml, Sigma-Aldrich), and brefeldin A (3 µg/ml), for 5 hours at 37°C, 5% CO_2_. The cells were then washed, fixed in 2% formaldehyde, incubated for 20 min in saponin buffer, and the expression of Th1 cytokines was assessed by staining of intracellular cytokines, and flow cytometry as described above.

### Detection of intermediates of NFκB signaling pathway by western blot

5×10^6^ BMM were seeded in 6-well cell culture plates, and incubated for 4 hours at 37°C, 5% CO_2_ to promote adherence. The cells were thereafter infected with *L. major* promastigotes using a 1∶15 ratio, and further incubated at 37°C, 5% CO_2_. At different time points (t = 0, t = 15 min, t = 30 min, and t = 1 h), lysates were prepared as follows: two different buffers were prepared, cytoplasmic cell fractionation buffer (10 mM HEPES, 10 mM KCl, 1.5 mM MgCl2, 0.34 M D-sucrose, 10% glycerin and 1 mM dithiothreitol (DTT), and nuclear cell fractionation buffer (3 mM ethylenediaminetetraacetic acid (EDTA), 0.2 mM ethylene glycol tetraacetic acid (EGTA), and 1 mM DTT). Directly prior to use, both buffers were supplemented with DTT (final concentration 0.5 mM), protease inhibitor cocktail (1∶100 dilution, Sigma-Aldrich), and Na_3_VO_4_ (final concentration 1 mM). At each time point, the stimulated cells were washed twice with cold PBS, and resuspended in 90 µl of ice-cold cytoplasmic cell fractionation buffer. 10 µl of 1% Triton X-100 in cytoplasmic cell fractionation buffer were added to the samples, and they were further incubated for 5 min on ice with gentle agitation. The samples were then centrifuged at 2000× g for 5 min at 4°C, and the supernatants were collected as cytoplasmic fraction, and stored at −20°C. The pellets were then washed with 100 µl of cytoplasmic cell fractionation buffer, and the samples were centrifuged again as described above. The supernatants were discarded, and the pellets were resuspended in 60 µl of nuclear cell fractionation buffer. The samples were further incubated for 30 min on ice. Then, they were sonicated on ice (Sonoplus, Bandelin, Berlin, Germany) using two cycles of 20 s each, with 40% of amplitude. The resulting suspensions were collected as nuclear fraction, and were stored at −20°C. The protein concentration of each sample was determined using a microplate bicinchoninic acid (BCA) protein assay kit (Thermo Scientific), following the manufacturer's instructions. 40 µg of protein from each sample were separated by SDS-PAGE (10% acrylamide gels), and transferred to poly(vinylidene difluoride; PVDF) membranes. The membranes were incubated overnight with primary Ab against the p65 subunit of NFκB (1: Santa Cruz, Dallas, USA, 2: Cell Signaling, Danvers, USA), MEK1/2, Lamin A/C (Cell Signaling), and Ctsb (R&D Systems, Minneapolis, USA). For detection, the membranes were incubated for 1 hour at RT with their corresponding horseradish peroxidase (HRP)-conjugated secondary Ab (Cell Signaling), and developed using a chemiluminescence kit (GE Healthcare, Munich, Germany). The membranes were then visualized using a FluorChem Q imager (Biozym Scientific, Oldendorf, Germany).

### Statistical analysis

Values are provided as mean ± standard deviations from at least 3 independent experiments. Statistical significance was determined by the unpaired 2-tail Student's *t* test (Microsoft Excel Software) comparing, for each treatment, the results from Ctsb^−/−^ or Ctsl^−/−^ cells with their WT counterparts.

## Results

### Cathepsins B and L are dispensable for the generation of functional BMM and BMDC

**Figure 1:**
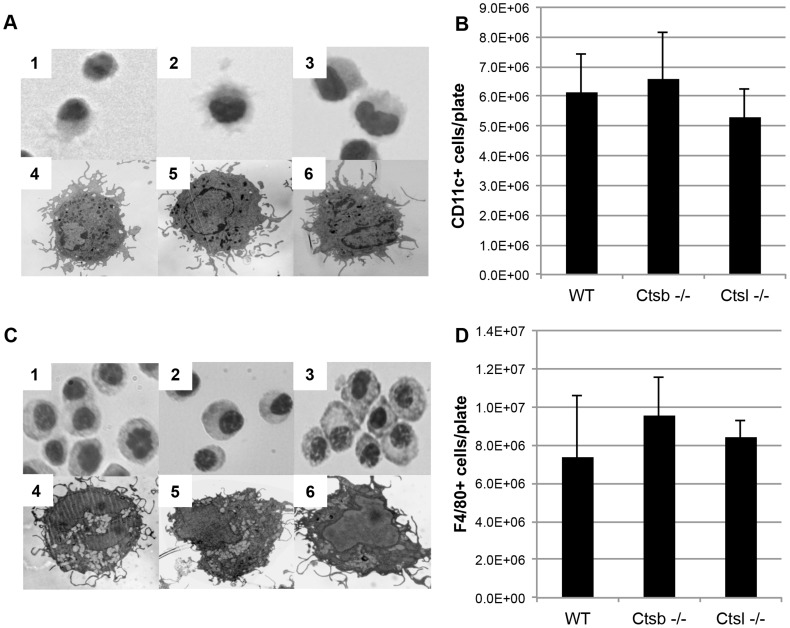
Comparable yields and phenotypes of BMDC and BMM generated from cathepsin B- and cathepsin L-deficient mice. BMDC from Ctsb^−/−^ and Ctsl^−/−^ mice present similar morphologies as WT BMDC. (A) Light microscopy pictures, using 40× magnification, of BMDC stained with Diff-Quik, 1: WT, 2: Ctsb^−/−^, 3: Ctsl^−/−^, and TEM pictures of BMDC, using a 4400× magnification, 4: WT, 5: Ctsb^−/−^, 6: Ctsl^−/−^. (B) No significant differences in the amount of CD11c^+^ cells generated per plate were found in Ctsb^−/−^ and Ctsl^−/−^ mice in comparison with WT mice. The results are expressed as mean ± SD of cells recovered per plate from 3 animals. (C) Similar morphologies found in BMM derived from WT and cathepsin-deficient mice (Diff-Quik staining 1: WT, 2: Ctsb^−/−^, 3: Ctsl^−/−^; TEM 4: WT, 5: Ctsb^−/−^ and 6: Ctsl^−/−^). (D) No significant differences were found in the amount of F4/80^+^ cells generated per plate from WT, Ctsb^−/−^ and Ctsl^−/−^ mice.

We used bone marrow stem cell progenitors from Ctsb^−/−^, Ctsl^−/−^ and WT (C57BL/6) mice to generate BMDC. At day 8 of culture with GM-CSF, the cells from all these mice displayed a typical myeloid immature DC morphology (Figure 1A, 1–6). These cells presented similar levels of CD11c expression, and comparable yields of CD11c^+^ cells were obtained (Figure 1B). Similarly, BMM generated from these cathepsin-deficient mice did not show any significant differences in morphology (Figure 1C, 1–6) and levels of F4/80 expression (Figure 1D) in comparison with BMM from WT mice.

**Figure 2:**
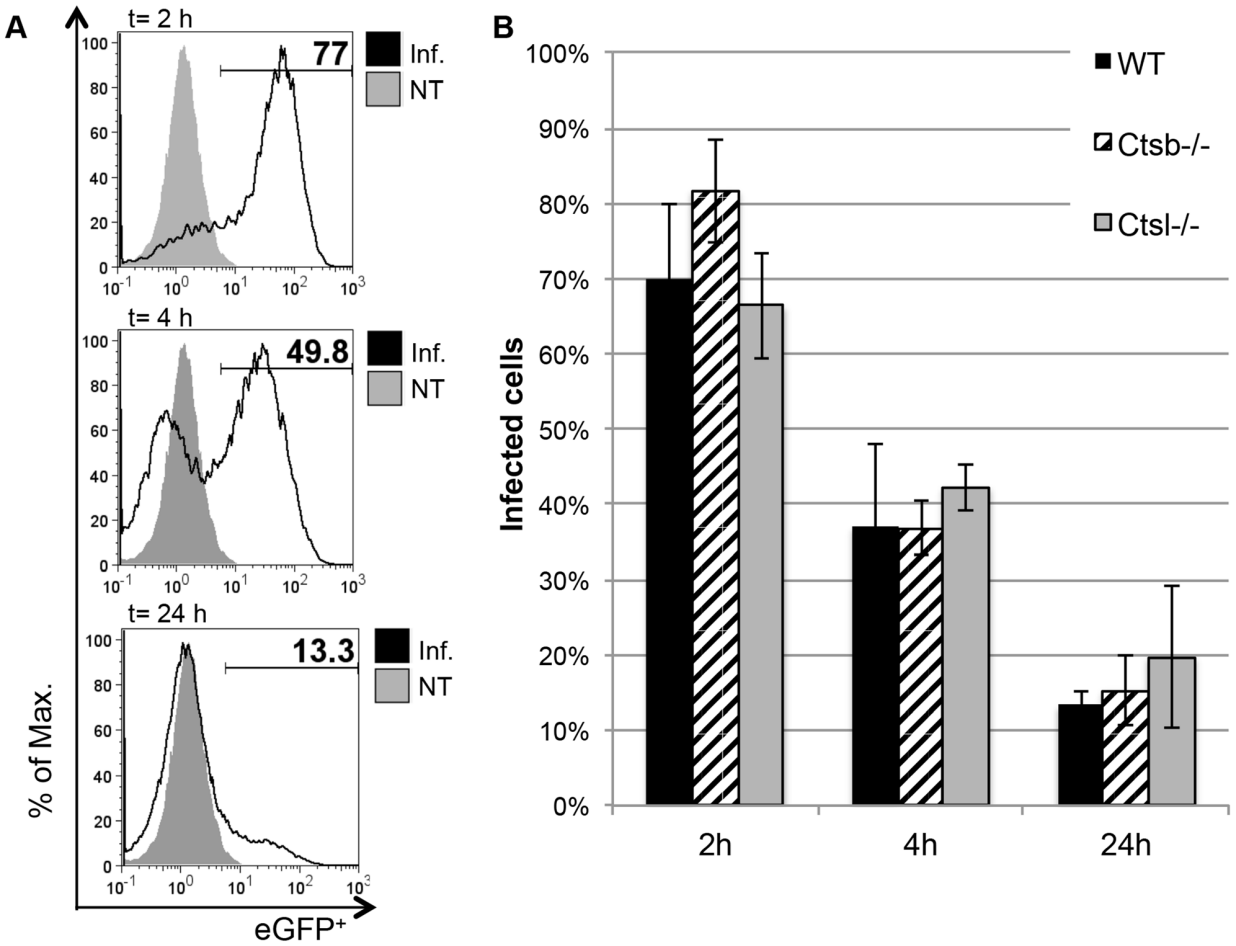
Comparison of *L. major* promastigote uptake and processing by BMDC from WT and cathepsin-deficient mice. (A) Representative histograms for WT BMDC (CD11c^+^-gated) infected for 2 hours with eGFP-tg *L. major* promastigotes, and further incubated for 4 and 24 hours in fresh medium. The percentage of eGFP^+^ cells was considered as percentage of remaining infected cells. (B) No significant differences between BMDC from WT and cathepsin-deficient mice were found in the uptake and processing of eGFP-tg promastigotes over the course of 24 hours. The results are expressed as mean ± SD of 3 independent experiments.

Next, eGFP-tg *L. major* promastigotes were used to analyze the kinetics of parasite uptake and processing by BMDC. After 2 hours of culture with *L. major* promastigotes at a parasite-to-BMDC ratio of 5 to 1, most of the WT cells (70%±9.8%) were infected with *L. major*, and rapidly processed the phagocytosed promastigotes (Figure 2A). At 24 hours after infection, only around 13% of the cells remained infected. BMDC from *Ctsb^−/−^* and *Ctsl^−/−^* mice showed no significant differences neither in the uptake of eGFP-tg *L. major* promastigotes nor in their kinetics for processing the parasites in comparison with WT BMDC (Figure 2B). These results indicate that cathepsins B and L are not relevant for the generation of BMM and BMDC, and that the capacity of the latter to phagocytize and process *L. major* promastigotes is not altered by the lack of cysteine cathepsins.

### Lack of cathepsin B and cathepsin L has no effect on intracellular parasites in macrophages

**Figure 3:**
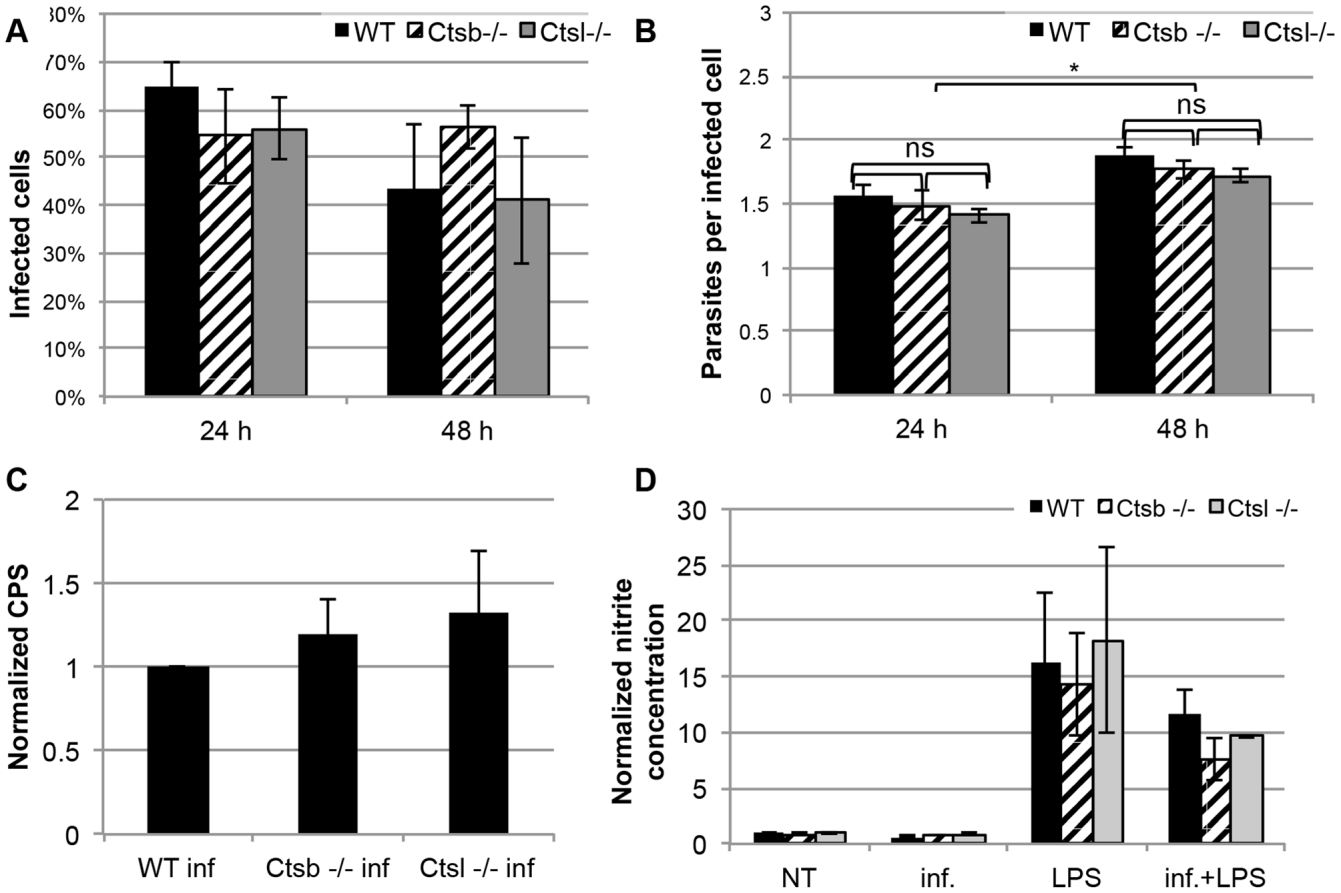
*L. major* promastigotes survive comparably in BMM from cathepsin B- and cathepsin L-deficient mice, and these BMM show similar levels of NO production in response to *L. major* and LPS. WT, Ctsb^−/−^ and Ctsl^−/−^ BMM were infected with eGFP-tg *L. major* promastigotes, and the percentage of infected cells 24 hours post infection (p.i.) and 48 hours p.i. was determined by fluorescence microscopy (A). No statistically significant differences were found in the amounts of infected cells between WT and cathepsin-deficient BMM. (B) As in (A) the number of parasites per infected cells was determined by fluorescence microscopy, and used as an indicator for parasite proliferation. Although no significant differences were found between WT and cathepsin-deficient BMM, each line showed significant differences in the counts of parasites per infected cell between 24 hours and 48 hours p.i. (C) Parasite proliferation determined by luminescence of Luc-tg *L. major* promastigotes within BMM at 48 hours p.i. Data are shown as counts per second (CPS). (D) NO production in supernatants from BMM 48 hours after infection with *L. major* and stimulation with LPS. Results are expressed as mean ± SD from 3 independent experiments.

We examined the survival of eGFP-tg *L. major* in macrophages. At 24 and 48 hours after infection, BMM from WT, *Ctsb^−/−^* and *Ctsl^−/−^* mice showed no significant differences in terms of percentage of infected cells, and average number of parasites per infected cell (Figures 3A and 3B, and Figure S1). To confirm these results, we also infected BMM from these mice with Luc-tg. *L. major* promastigotes, and measured the luminescence produced after addition of a luciferin substrate as a read-out for intracellular parasites. Again, no significant differences were found between WT BMM and cathepsin-deficient-BMM (Figure 3C).

In addition, we measured the nitrite concentrations in supernatants of BMM infected with *L. major* in the presence or absence of LPS, and in response to LPS alone (Figure 3D). We found that 48 hours of infection with *L. major* promastigotes alone did not result in higher NO production compared to non-infected cells from WT, *Ctsb^−/−^* and *Ctsl^−/−^* BMM. Furthermore, we found no significant differences in nitrite levels in the supernatants of these cells neither after stimulation with LPS alone nor after infection with *L. major* promastigotes and further stimulation with LPS. These results show that cathepsin B and cathepsin L are dispensable for the control *in vitro* of *L. major* in BMM, and that absence of either of them does not affect the capacity of BMM to produce NO in response to neither *L. major* nor LPS.

### Cathepsin B-deficient DC express higher levels of MHC class II molecules in response to *L. major* promastigotes

**Figure 4:**
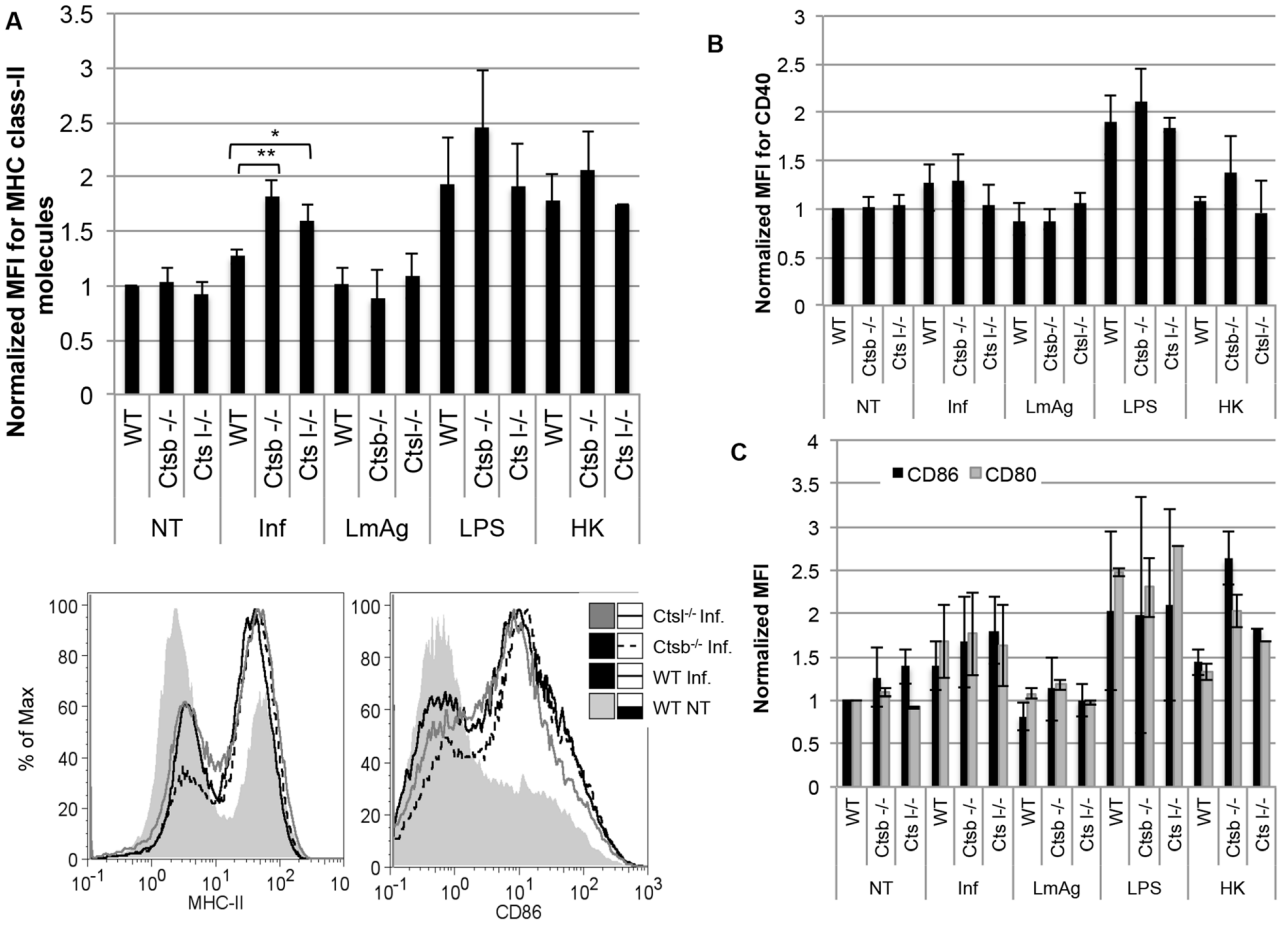
BMDC from cathepsin B-deficient mice express higher levels of MHC class II molecules in comparison with BMDC from cathepsin L-deficient and WT mice, but no differences were observed in the expression of co-stimulatory molecules. The expression of MHC class II molecules, CD40, CD86 and CD80 was measured by flow cytometry in BMDC in response to infection with *L. major* promastigotes (Inf), LmAg, heat-killed parasites (HK), or LPS. (A) Average MFI of MHC class II molecules, normalized to non-treated (NT) WT BMDC. (B) Average MFI of CD40, normalized WT NT BMDC. (C) Average MFI of CD80 and CD86, normalized WT NT BMDC. MFI values are expressed as mean ± SD of 4 independent experiments. Statistical significance was assessed between WT BMDC and Ctsb^−/−^ BMDC, and between WT BMDC and Ctsl^−/−^ BMDC for every single treatment, * p<0.05, *** p<0.005.

Upon encounter with pathogens, immature DC become activated and mature, up-regulating the expression of the antigen-presenting molecules MHC class II as well as co-stimulatory molecules such as CD86, CD80 and CD40 [37]. We analyzed the maturation profile of BMDC from WT, *Ctsb^−/−^* and *Ctsl^−/^* ^−^ mice 24 hours after uptake of *L. major* promastigotes. We found that the expression of MHC class II molecules was greatly enhanced in BMDC from *Ctsb^−/−^* mice and, to a lesser extent, in BMDC from *Ctsl^−/−^* mice in response to *L. major* compared to WT BMDC (Figure 4A). The expression of the co-stimulatory molecules CD40, CD86, and CD80, on the other hand, was comparable among WT, *Ctsb^−/−^* and *Ctsl^−/−^* BMDC (Figures 4B and 4C). In addition, we tested the expression of MHC class II and co-stimulatory molecules of BMDC pre-incubated with CLIK148 and CA074Me, a modified form of CA074 with increased cell permeability. BMDC treated with CA074Me showed an up-regulation of MHC class II molecules in response to *L. major* promastigotes higher than that observed for BMDC treated with CLIK148 or DMSO. On the other hand, we found no effect on CD86 expression, similar to the results obtained with the use of cathepsin-deficient cells (Figure S2 A and B).

These higher expression levels of MHC class II molecules in *Ctsb^−/−^* BMDC appeared to be a specific response to living parasites, since stimulation of *Ctsb^−/−^* BMDC with LmAg or HK parasites did not result in enhanced expression levels of MHC class II molecules and co-stimulatory molecules in comparison with WT and *Ctsl^−/−^* mice (Figure 4). In addition, no significant differences in the expression of co-stimulatory and MHC class II molecules were found in WT and cathepsin-deficient BMDC upon stimulation with LPS. The activation by inflammatory stimuli is known to recruit cathepsins B, L and S to late endosomes [38], [39], and, therefore, it is possible that different stimuli would lead to different profiles of active cathepsins for antigen processing.

Our results demonstrate that *L. major*-stimulated *Ctsb^−/−^* BMDC, on the basis of MHC class II expression, display an enhanced maturation compared to WT and *Ctsl^−/^* ^−^ BMDC in response to *L. major* promastigotes. On the other hand, no significant differences in the expression of the co-stimulatory molecules CD86, CD80, and CD40 were observed. This effect was not elicited by stimulation with LmAg, HK parasites, or with LPS.

### Cathepsin B-deficient DC and macrophages express IL-12 in response to *L. major* promastigotes

**Figure 5:**
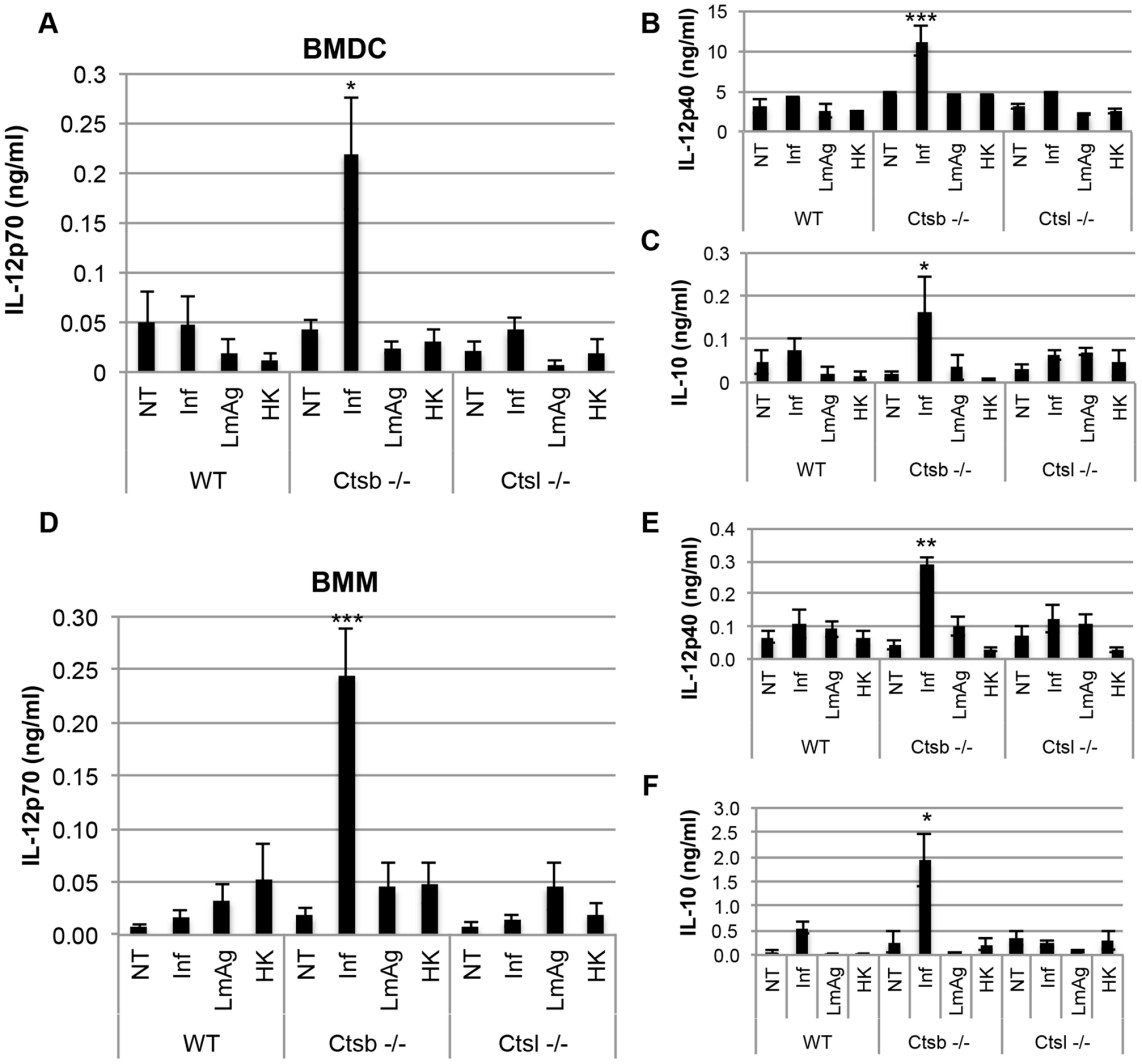
BMDC and BMM from cathepsin B-deficient mice express higher levels of IL-12 in response to *L. major* than cells derived from WT and cathepsin L-deficient mice. (A) IL-12p70 in supernatants from non-treated BMDC(NT), BMDC infected (Inf) with *L. major* promastigotes at 48 hours p.i., BMDC stimulated with parasite lysate (LmAg), or with heat-killed parasites (HK), for 48 hours. (B) IL-12p40 and (C) IL-10 concentration in supernatants of BMDC at 48 hours p.i., or stimulation with LmAg or HK parasites. (D) IL-12p70 production in supernatants from non-treated BMM (NT), BMM infected (Inf) with *L. major* promastigotes at 48 hours p.i., and BMM stimulated for 48 hours with LmAg or HK parasites. (E) IL-12p40 and (F) IL-10 concentration in supernatants of BMM at 48 hours p.i., or stimulation with either LmAg or HK parasites. The results are expressed as mean ± SD of 5 independent experiments. For each experimental group (NT, Inf, LmAg and HK), statistical significance was estimated between WT and Ctsb^−/−^ cells, and between WT and Ctsl^−/−^ cells, * p<0.05, **p<0.01, *** p<0.005.

Another important signal that BMDC use to instruct Th cell polarization is cytokine production. We analyzed the concentrations of the cytokines IL-12p70, IL-12p40, IL-10, IL-6 and TNF-α in supernatants of BMDC 48 hours after infection with *L. major* promastigotes, or stimulation with LmAg. We found a significant increase in both IL-12p70 and IL-12p40 levels in *Ctsb^−/−^* BMDC in response to *L. major* in comparison with WT and *Ctsl^−/−^* BMDC (Figures 5A and 5B). We also found that IL-10 expression was enhanced in *Ctsb^−/−^* BMDC (Figure 5C), resembling the production of IL-10 in response to an up-regulation of IL-12 observed in DC after stimulation with LPS, a Th1 inducer. Moreover, the IL-12 up-regulation required living parasites, since stimulation of BMDC with LmAg or HK parasites did not induce higher levels of IL-12 production. In addition, we found no differences in IL-6 and TNF-α production between WT and cathepsin-deficient BMDC in response to *L. major* parasites (Figure S3). In contrast, *Ctsb−/−* BMDC presented an impaired IL-12p70 expression in response to CpG in comparison with WT BMDC, which reflects the importance of Ctsb in Toll-like receptor 9 (TLR9) signaling (Figure S4). The IL-12 up-regulation in response to *L. major* observed with *Ctsb^−/−^* BMDC could not be replicated using the inhibitor CA074Me (Figure S2, C), and this inhibitor caused a dose-dependent decrease in IL-12p70 in LPS-stimulated cells (Figure S2, D). However, when BMDC from BALB/c and C57BL/6 mice were pre-treated with the peptide-based cathepsin B inhibitor ZRLR, they up-regulated their expression of IL-12p70 in response to *L. major* promastigotes resulting in levels comparable to those observed in Ctsb^−/−^ BMDC (Figure S5, A). Pre-treatment of Ctsb^−/−^ BMDC with ZRLR did not cause significant differences in the expression of IL-12 in response to *L. major* in comparison with DMSO pre-treated Ctsb^−/−^ BMDC. In addition, we found a very similar pattern of increased IL-12p70, IL-12p40 and IL-10 expression in *Ctsb^−/−^* BMM in comparison with WT and *Ctsl^−/−^* BMM (Figures 5D to F). It should be noticed that the levels of IL-12p40 were considerably lower in BMM than in BMDC.

**Figure 6:**
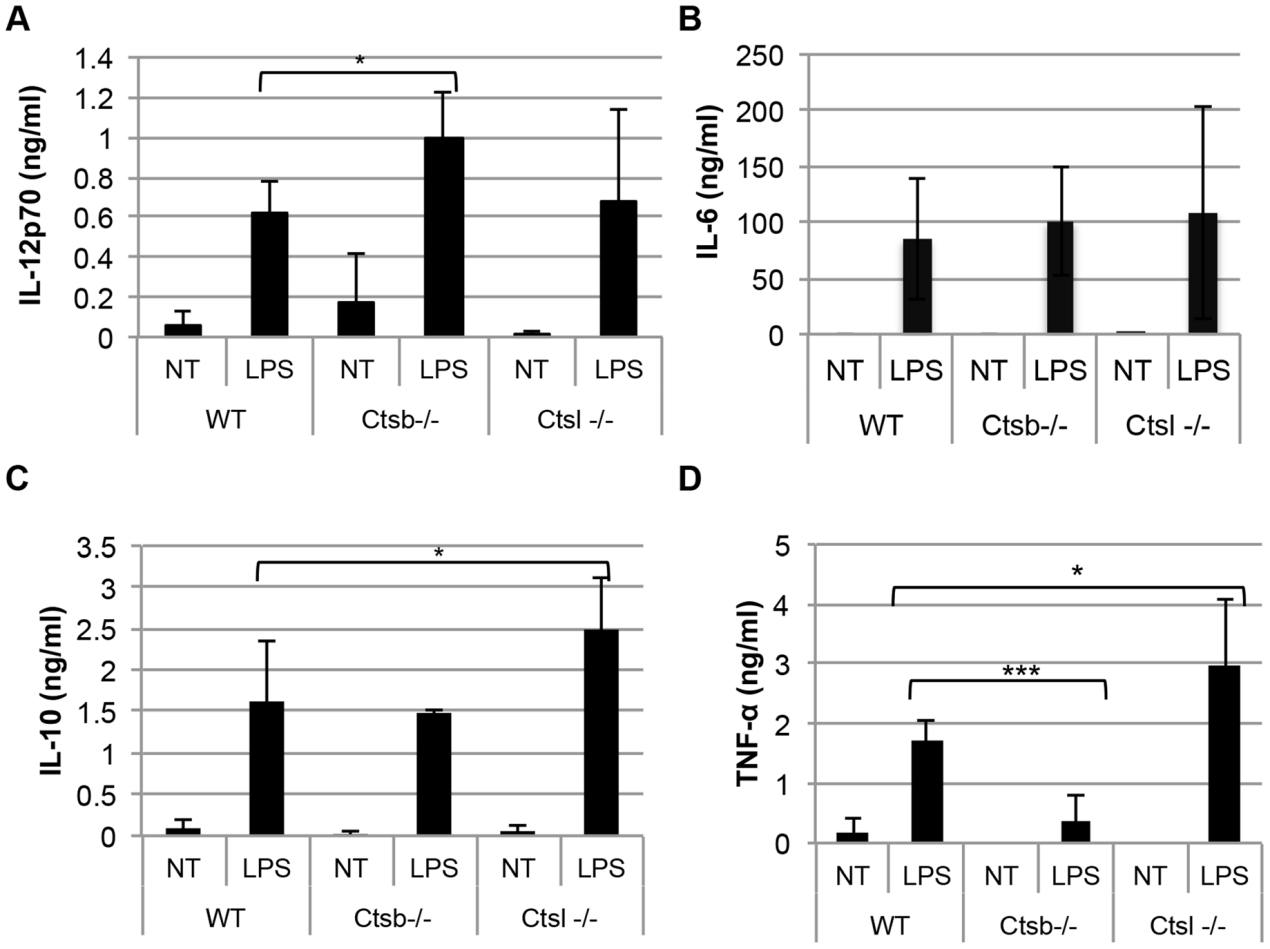
BMDC from cathepsin B-deficient mice express higher levels of IL-12 in response to LPS than cells from WT and cathepsin L-deficient mice. Concentration of different cytokines in supernatants from non-treated BMDC (NT) or LPS-stimulated BMDC (LPS, 1 µg/ml) after 24 hours: (A) IL-12p70, (B) IL-6, (C) IL-10, and (D) TNF-α. The results are expressed as mean ± SD of 5 independent experiments. The statistical significance in each treatment was assessed between WT and Ctsb^−/−^ BMDC, and between WT and Ctsl^−/−^ BMDC. * p<0.05, **p<0.01, *** p<0.005.

**Figure 7:**
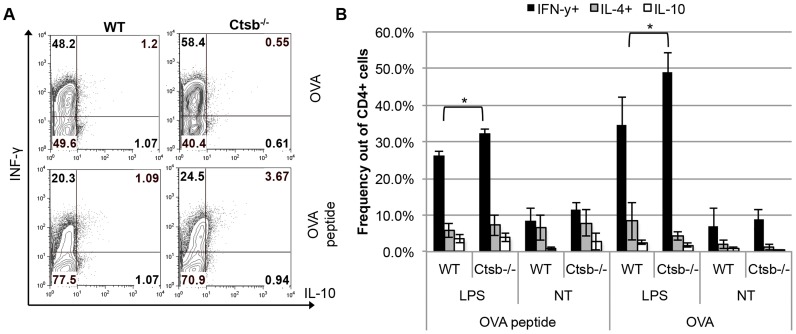
Th1 polarization of OT-II CD4^+^ naïve T cells by BMDC from WT C57BL/6 and Ctsb^−/−^ mice. Isolated CD4^+^ CD25^−^ T cells from OT-II mice were co-cultured with BMDC generated from WT C57BL/6 and Ctsb^−/−^ mice in the presence of LPS as a stimulus and OVA peptide (327–339) or ovalbumin (OVA) as antigens. A) Zebra plots from one representative experiment. B) Average percentages of IFN-γ, IL-4, or IL-10^+^ CD4^+^ T cells from 3 independent experiments ± SD. * p<0.05.

**Figure 8:**
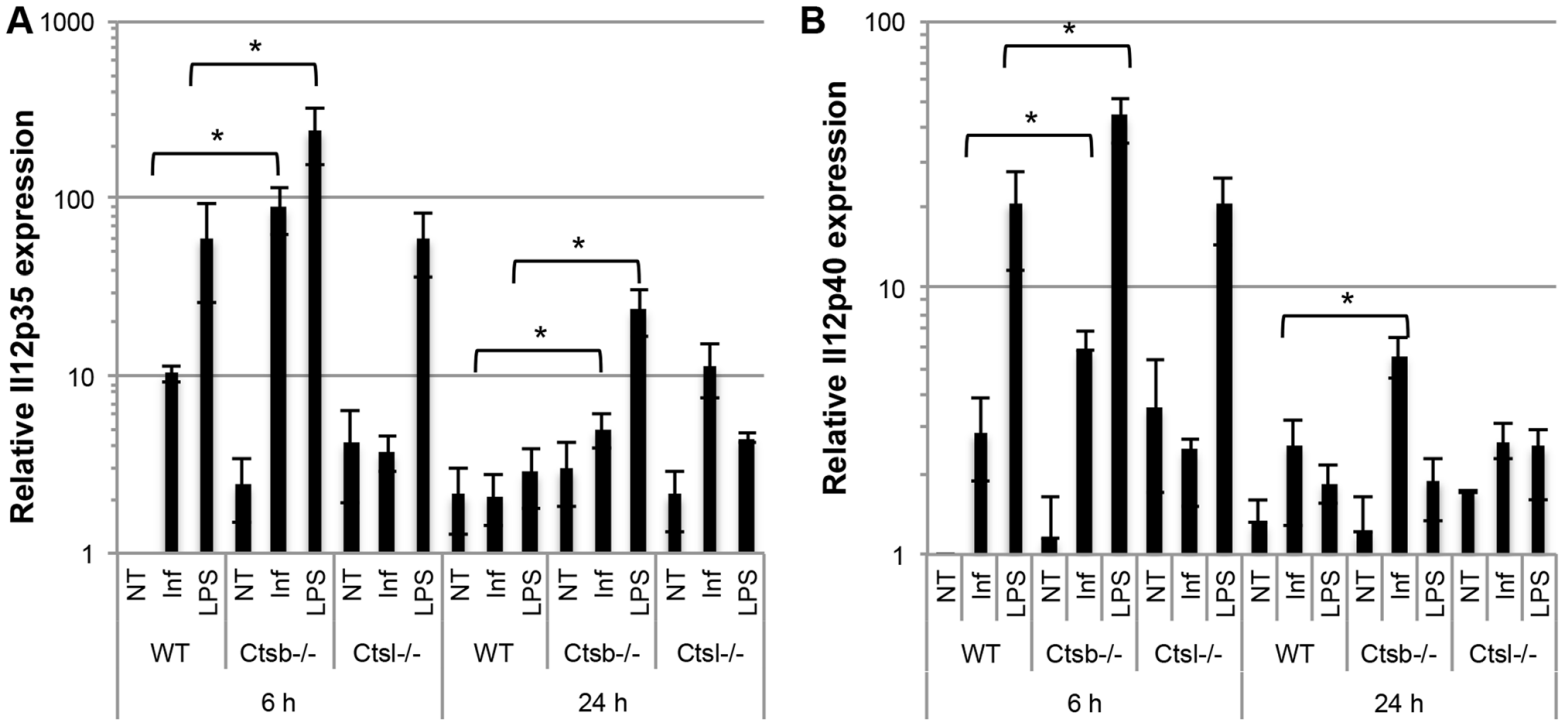
IL-12 is up-regulated at the transcriptional level in BMM from cathepsin B-deficient mice in response to *L. major* infection and LPS stimulation. Relative expression levels of (A) IL-12p40 and (B) IL-12p35 transcripts in mRNA from BMM at 6 hours or 24 hours p.i. with *L. major* promastigotes or LPS stimulation. Non-treated (NT) BMM from each mouse line were used as negative controls. The expression levels were estimated using the 2^− ΔΔC^_T_ method, using WT NT BMM at t = 6 hours as a reference. The results are shown as mean ± SD of 4 independent experiments, * p<0.05.

Moreover, *Ctsb^−/−^* BMDC stimulated with LPS also produced slightly more IL-12p70 and IL-12p40, but not IL-10, compared to WT and *Ctsl^−/−^* BMDC. However, TNF-α expression was greatly impaired in *Ctsb^−/−^* BMDC, and no significant differences in IL-6 production among WT, *Ctsb^−/−^* and *Ctsl^−/−^* were found (Figure 6). This effect in IL-12 expression was also found in BMDC from BALB/c and C57BL/6 mice pre-treated with ZRLR (Figure S5, B to D). When co-cultured with naïve T cells from OT-II mice, we found that Ctsb^−/−^ BMDC having LPS as a maturation stimulus resulted in higher frequencies of IFN-γ^+^ T cells, but not of IL-4^+^ T cells, indicating a Th1 polarization (Figure 7). Furthermore, the enhanced IL-12 production in response to *L. major* and LPS that we observed was found also at the transcriptional level, since *Ctsb^−/−^* BMM presented an up-regulation in the expression of *Il12p35* and *Il12p40* in response to both stimuli (Figure 8).

Next, we investigated if this up-regulation of IL-12 was dependent on the NF-κB signaling pathway by assessing the translocation of the p65 subunit from the cytoplasm to the nucleus (Figures S6 and S7). We tested separately two different monoclonal antibodies to detect the NF-κB p65 subunit by Western Blot, and used for analysis the protein bands with the expected molecular weight (65 kDa). We found that the different levels of p65 for all the treatments in nuclear fractions from WT and Ctsb^−/−^ had no statistical significance. We should point out that we found with both antibodies multiple protein bands with molecular weights other than 65 kDa. In particular, we observed a non-identified protein with a molecular size around 30 kDa in WT LPS-stimulated BMM but not in LPS-stimulated *Ctsb^−/−^* BMM. While these results suggest that NF- κB is not responsible of the *Ctsb^−/−^* -mediated regulation of IL-12, more experiments with different approaches would be needed to confirm this observation.

Altogether, our results show that *Ctsb^−/−^* BMDC and BMM presented a significant up-regulation of the Th1 promoter cytokine IL-12 in response to *L. major* and LPS, in comparison with WT and *Ctsl^−/−^* cells. This effect could be replicated using the peptide-based cathepsin B inhibitor ZRLR in BMDC from mouse strains susceptible and resistant to *L. major*. Furthermore, the observed up-regulation of IL-12 was present already at the transcriptional levels, and Ctsb^−/−^ BMDC induced higher frequencies *in vitro* of Th1-polarized T cells.

## Discussion

In the present study, we investigated the differences in the signals that BMDC use to instruct Th cell polarization, *i.e.*, antigen presentation, expression of co-stimulatory molecules and cytokines, as a response to *L. major* promastigotes in the absence of Ctsb and Ctsl. In addition, we analyzed the impact of the lack of these proteases on the proliferation of *L. major* in infected BMM. We found that *Ctsb^−/−^* BMDC express higher levels of MHC class II molecules and of IL-12 in response to *L. major* promastigotes, and that this up-regulation of IL-12 expression was also present in BMM. These results indicate a novel role for Ctsb in the regulation of cytokine expression in response to *L. major*.

Stem cell progenitors from *Ctsb^−/−^* and *Ctsl^−/−^* mice are able to generate BMDC and BMM with comparable yields and phenotypes as WT mice. These cells presented similar rates of parasite uptake, and BMDC showed similar kinetics of parasite processing. Moreover, parasite survival was similar in cathepsin-deficient and WT BMM. These results indicate that the up-regulation in MHC class II molecules and IL-12 expression that we observed was not due to differences in the parasite load between WT, *Ctsb^−/−^* and *Ctsl^−/−^* BMDC.

DC present an incomplete maturation after uptake of *L. major* promastigotes [40] and *L. amazonensis* [41]. Previous studies using the Ctsb-selective inhibitor CA074 and the Ctsl-specific inhibitor CLIK148 showed drastic changes in the Th cell response of mice infected with *L. major* [25], [28], [42], and it was hypothesized that these effects resulted from differences in antigen processing. Later reports showed that cathepsin S is indispensable for the degradation of the invariant chain in antigen-presenting cells [43]–[45], while Ctsl was relevant for the processing of antigens only in cortical thymic epithelial cells [46]. Ctsb and cathepsin D (Ctsd) were shown to be dispensable for the maturation of MHC class II molecules and the presentation of several antigens [31]. However, this study also reported that splenocytic antigen-presenting cells from Ctsb- or Ctsd-deficient mice were actually more efficient to present certain antigens to T-cell hybridomas, in agreement with reports using different inhibitors with murine splenocytes [47] and primary human DC [48]. The latter study described the use of the peptide-based cathepsin inhibitor ZRLR which was shown to have superior specificity towards Ctsb compared to CA074Me. Deussing et al. suggested that some antigenic determinants may present different degrees of susceptibility to degradation by cathepsins before being able to bind to MHC class II molecules, and, therefore, would benefit from the absence of Ctsb or Ctsd [31]. We found higher levels of MHC class II molecules in *Ctsb^−/−^* BMDC than in WT and *Ctsl^−/−^* BMDC in response to *L. major*. Similar results were found with the use of the inhibitor CA074Me, while no significant differences were found when we used LmAg or heat-killed parasites as stimulus. The different results observed with promastigote- and LmAg-mediated stimulation could reflect the interaction of the living parasite with the host cell, and the differences in uptake mechanism and subsequent processing [47], [48]. Stimulation with heat-killed parasites led to comparable levels of MHC class II molecules and co-stimulatory molecules as observed with infected BMDC. However, we found no significant differences between WT and cathepsin-deficient BMDC. This could indicate that the higher levels of MHC class II molecules in infected Ctsb−/− BMDC in comparison with WT BMDC are related to the active manipulation that the living parasite exerts in its host cell. Incomplete BMDC maturation, such as after stimulation with *Trypanosoma brucei* antigens, has been shown to induce activation of genes correlating with the induction of Th2 polarization [49]. In contrast, higher levels of antigen presented [50] are associated with induction of Th1 responses.

Upon *in vitro* infection with *Leishmania* promastigotes, BMDC present poor cytokine expression [51]. We found that *Ctsb^−/−^* BMDC were able to express significantly higher levels of IL-12 (both p70 and p40 forms) than WT and *Ctsl^−/−^* BMDC in response to *L. major* promastigotes. We did not detect significant differences in IL-6 and TNF-α, which would indicate that the observed effect was not a generalized up-regulation of cytokine expression, but a rather specific mechanism. Likewise, infection of BMM with *L. major* promastigotes induces poor cytokine expression [17], [19]. *Ctsb^−/−^* BMM also presented a significant increase in IL-12 expression, with similar levels of IL-12p70 as *Ctsb^−/−^* BMDC, although the up-regulation of IL-12p40 was not as high. Pompei et al. reported a differential release of IL-12 in BMDC and BMM in response to *Mycobacterium tuberculosis*, and suggested that this was dependent upon the level of engagement of different TLR, particularly TLR9 in BMDC [52]. TLR9 requires processing by endosomal cathepsins to initiate signaling [53], [54]. In agreement with Matsumoto et al. [53], we observed a great impairment in IL-12 expression in *Ctsb^−/−^* BMDC upon CpG stimulation. Therefore, the up-regulation in IL-12 expression by *Ctsb^−/−^* BMDC and BMM that we observed here is independent from TLR9 signaling.

In addition, we tested the response of *Ctsb^−/−^* and *Ctsl^−/−^* BMDC to LPS, which is recognized by TLR4. *Ctsb^−/−^* BMDC stimulated with LPS did not show a significantly higher expression of MHC class II molecules in comparison with WT BMDC or *Ctsl^−/−^* BMDC but they did display higher levels of IL-12. Moreover, the expression of TNF-α was greatly impaired in *Ctsb^−/−^* BMDC, in agreement with Ha et al. [55] who reported that LPS-treated BMM secrete significantly less TNF-α in response to LPS upon lack of Ctsb, due to an accumulation of TNF-α-containing vesicles that could not reach the plasma membrane. Schotte et al. reported an impairment of cytokine production in macrophages stimulated with LPS upon treatment with the cathepsin B inhibitor z-FA.fmk [56]. Our results with *Ctsb^−/−^* BMDC and BMM do not show an inhibition of IL-12 expression but rather an enhancement. We obtained similar results using CA074Me, the methyl ester form of CA074. Upon uptake by the cell, CA074Me is hydrolyzed to CA074, but if this hydrolysis is incomplete, inhibition of other cysteine proteases besides Ctsb takes place [57]. In contrast, pre-treatment of BMDC from susceptible BALB/c or resistant C57BL/6 mice with ZRLR induced IL-12 expression levels comparable to those observed with *Ctsb^−/−^* BMDC. Although we did not have Ctsb^−/−^ mice on a BALB/c background available, these results suggest that the observed role of Ctsb in *L. major* infection would be independent of the mouse strain.

Upon infection with *L. major* promastigotes, *Ctsl^−/−^* BMDC and BMM did not present significant differences in the production of cytokines in comparison with WT cells. However, they produced higher levels of IL-10 and TNF-α, but not IL-12, in response to LPS. These results alone would not explain the observations made by Onishi et al. [58], where use of the cathepsin L inhibitor CLIK148 caused a Th2-like immune response to *L. major* in otherwise resistant mice. Again, it should be kept in mind that CLIK148 can also inhibit other cathepsins, including C, K, and S [59], which could have contributed to this response.

Altogether, while previous studies hypothesized that lack of Ctsb or Ctsl activities during *L. major* infection would lead to changes in Th cell polarization due to differences in antigen presentation [25], [28], [42], our results indicate that *Ctsb^−/−^* BMDC up-regulate two of the three types of signals used for instructing Th cell polarization: expression of MHC class II molecules and cytokine expression. Thus, these cells exhibit a “pro-Th1”-like profile. Moreover, co-culture of purified naïve CD4^+^ T cells with Ctsb^−/−^ BMDC resulted in a higher frequency of Th1-polarized T cells compared to WT BMDC. To the best of our knowledge, the present study is the first indicating a new role of Ctsb as a regulator of cytokine expression in response to *L. major*. Future work will focus on the implications of these effects *in vivo*, considering the infection of Ctsb^−/−^ animals, as well as in transfer experiments of Ctsb^−/−^ BMDC into WT animals.

In which way could Ctsb influence cytokine production in BMDC and BMM? Ben-Othman et al. reported that *L. major* parasites induce macrophage tolerance by a process involving MAPK and NF-κB pathways of the host [60]. These pathways, although initially activated by exposure to the parasite, become silenced when the infection is firmly established, rendering the cell unresponsive to further stimulation with LPS [61]. This silencing has been attributed to different virulence factors, including surface phosphoglycans [16], [62], the metalloprotease GP63 [63], and cysteine proteases from *L. mexicana* [21]. It is possible that in the absence of Ctsb, one or more of these key signaling pathways are no longer silenced b*y L. major* promastigotes. This would open a range of new questions regarding the involvement of Ctsb in *L. major* infection, *e.g.*, whether Ctsb interacts directly with the parasites, contributing to processing or activation of one or more virulence factors, or whether Ctsb directly interferes with any intermediate of key signaling pathways, such as NF-kB and MAPK. While Ctsb^−/−^ BMDC and BMM were able to up-regulate IL-12 in response to *L. major* and LPS, IL-6 was not regulated. IL-6 transcription has been shown to depend on NF-κB [64]. Therefore, we hypothesize that the molecular mechanism behind the involvement of *Ctsb^−/−^* in the expression of IL-12 would not be shared by IL-6. Our results suggest that the regulation of IL-12 expression by Ctsb is not NF-κB-dependent, although further work is necessary to confirm this observation and to explore the interaction of Ctsb with other candidate signaling pathways. In addition, finding the location of these interactions would be a key to further understand the mechanisms underlying these processes, e.g., whether proteolytic processing of signaling intermediates takes place in the cytoplasm, or whether cleavage of transcription factors by Ctsb occurs in the nuclear space, as described in thyroid carcinoma cells [65].

The concept of “protease signaling” has gained increasing attention in different research fields [66], especially in the context of therapeutic applications. Yet, more research is needed in order to understand the interplay between proteolytic networks and other signaling pathways. On the basis of the present study, we propose a novel role for cathepsin B during *L. major* infection: in addition to its involvement in antigen presentation, it is also a regulator of cytokine expression. It is tempting to speculate that pharmacological inhibition of cathepsin B may improve the Th1-mediated clearance of *L. major*.

## Supporting Information

Figure S1 **Proliferation of** ***L. major*** **in BMM from WT, Ctsb^−/−^ and Ctsl^−/−^ BMM.** (A) Frequency of infected BMM harboring 1, 2 or 3 or more parasites at 24 hours and 48 hours p.i. (B). Percentage of parasites per infected BMM at 24, 48 and 72 hours p.i. Although no significant differences were found between WT and *Ctsb^−/−^* BMM, each line showed significant differences in the counts of parasites per infected cell between 24 hours and 48 hours p.i., and between 24 hours and 72 hours p.i. (C) Frequency of infected BMM harboring 1, 2, 3 or more parasites at 24, 48 and 72 hours p.i. The results are shown as mean ± SD of 3 independent experiments.
(TIF)

Figure S2 **Effect of CA074Me and CLIK148 on BMDC in response to** ***L. major*** **promastigotes, and to LPS.** BMDC were pre-incubated with the cathepsin B inhibitor CA074Me (10 µM), the cathepsin L inhibitor CLIK148 (10 µM), or an equivalent volume of DMSO for 4 hours, followed by infection with *L. major* promastigotes. The levels of MHC class II molecules (A) and CD86 (B) were determined by flow cytometry. (C) Concentration of IL-12p70 in the supernatants of BMDC pre-incubated with CA074Me and infected with *L. major* promastigotes. (D) Measurement of IL-12p70 in supernatants of BMDC stimulated with LPS in the presence of different concentrations of CA074Me. The results are shown as mean ± SD of 3 independent experiments. The statistical significance in infected cells in (A), (B) and (C) was estimated between BMDC pre-incubated with DMSO and CA074Me, and between BMDC pre-incubated with DMSO and CLIK148, * p<0.05. The statistical significance in (D) was calculated for each CA074Me concentration against LPS-stimulated BMDC pre-incubated with DMSO. * p<0.05, *** p<0.005.
(TIF)

Figure S3 **BMDC and BMM from WT and cathepsin-deficient mice express similar levels of IL-6 and TNF-α in response to** ***L. major*** **and LmAg.** (A) TNF-α in supernatants from non-treated BMDC (NT), BMDC infected (Inf) with *L. major* promastigotes at 48 hours p.i. and BMDC stimulated with parasite lysate (LmAg) or heat-killed parasites (HK) for 48 hours. (B) IL-6 concentration in supernatants of BMDC at 48 hours p.i. (C) TNF-α in supernatants from non-treated BMM (NT), BMM infected (Inf) with *L. major* promastigotes at 48 hours p.i. and BMM stimulated with LmAg or HK for 48 hours. (D) IL-6 concentration in supernatants of BMM at 48 hours p.i. The results are expressed as mean ± SD of 3 independent experiments. For each treatment (NT, Inf, LmAg, and HK), statistical significance was assessed between WT and Ctsb^−/−^ cells, and between WT and Ctsl^−/−^ cells, and in all cases no statistical significance was found (p>0.05).
(TIF)

Figure S4 **IL-12p70 expression in response to CpG is impaired in BMDC from cathepsin B-deficient mice.** IL-12p70 was measured by ELISA in supernatants of non-treated (NT) or CpG-treated cells (25 µg/ml CpG, 24 hours stimulation). For each treatment, the statistical significance was calculated between WT and Ctsb^−/−^ BMDC, and WT and Ctsl^−/−^ BMDC. *p<0.05, ***p<0.005.
(TIF)

Figure S5 **Expression of IL-12 in BMDC of BALB/c and C57BL/6 mice in response to different stimuli after inhibition of cathepsin B with ZRLR.** (A) Measurement of IL-12p70 in supernatants from BMDC pre-incubated with 10 µM ZRLR, 10 µM CA074Me or DMSO, washed, and subsequently exposed to *L. major* promastigotes for 48 h. (B) Measurement of IL-12p70 by ELISA in supernatants of BMDC from BALB/c and C57BL/6 mice after 24 hours of stimulation with LPS in the presence of ZRLR or DMSO. The bars represent the average results from 3 independent experiments ± SD. IL-12(p40/p70) additionally was measured by intracellular staining. (C) MFI for IL-12(p40/p70); the bars represent the average MFI values from 3 independent experiments, normalized to the MFI values of NT DMSO C57BL/6 ± SD. (D) IL-12(p40/p70) histograms from one representative experiment.
(TIF)

Figure S6 **Measurement of NFκB (p65 subunit) in nuclear and cytoplasmic extracts by western blot.** Nuclear (N) and cytoplasmic (C) extracts were prepared from WT and Ctsb^−/−^ BMM at different time points after infection with *L. major* promastigotes or stimulation with LPS. (A) Quantification of NFκB (p65 subunit) by Western Blot, represented as arbitrary units (AU) relative to the measurements in WT BMM NT at t = 0 min. The bars represent the average result from 3 independent experiments ± SD. For each treatment, no statistical significance was found between samples from WT and Ctsb^−/−^ BMM. B) Representative immunoblots from one experiment including samples at t = 0 and t = 15 min. Multiple bands were detected independently using two different antibodies against NFκB (p65 subunit) 1: from Santa Cruz, 2: from Cell Signaling, however only those with an apparent molecular weight of 65 kDa (black arrows) were considered for the analysis in (A). The expression levels of MEK and Lamin A/C were used as loading controls for cytoplasmic and nuclear extracts, respectively.
(TIF)

Figure S7 **Measurement of NFκB (p65 subunit) in nuclear and cytoplasmic extracts by western blot (continuation of Figure S6).** Representative immunoblots from one experiment, same as shown in Figure S6 B, including samples at t = 30 min and t = 60 min.
(TIF)
