# Supplementary figures and images for: Cathepsin B in Antigen-Presenting Cells Controls Mediators of the Th1 Immune Response during Leishmania major Infection
Source: PLoS Negl Trop Dis. 2014 Sep 25;8(9):e3194. doi: 10.1371/journal.pntd.0003194 (PMC4177854; doi:10.1371/journal.pntd.0003194)

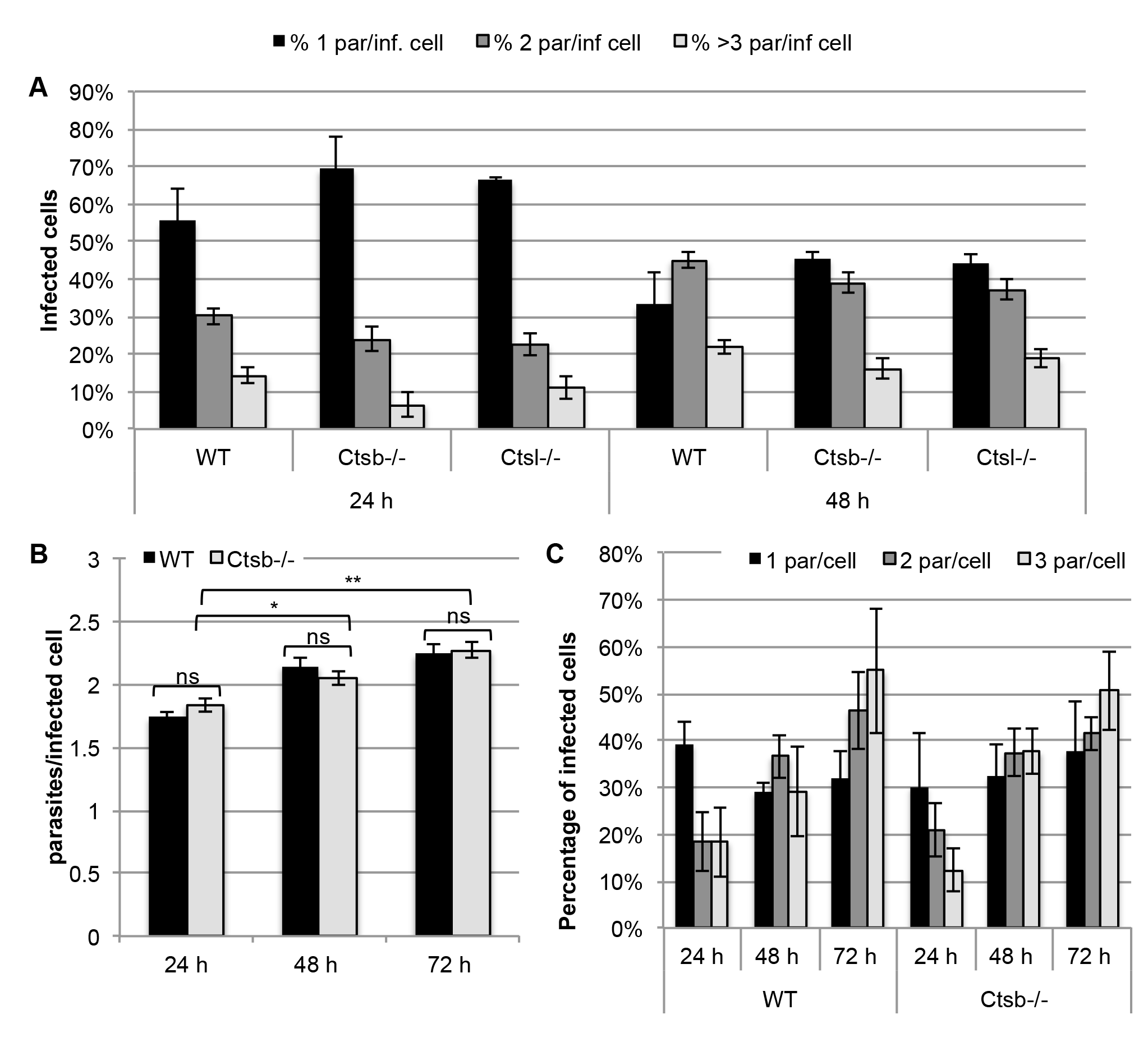

Supplement: Figure S1 — Proliferation of L. major in BMM from WT, Ctsb−/− and Ctsl−/− BMM. (A) Frequency of infected BMM harboring 1, 2 or 3 or more parasites at 24 hours and 48 hours p.i. (B). Percentage of parasites per infected BMM at 24, 48 and 72 hours p.i. Although no significant differences were found between WT and Ctsb−/− BMM, each line showed significant differences in the counts of parasites per infected cell between 24 hours and 48 hours p.i., and between 24 hours and 72 hours p.i. (C) Frequency of infected BMM harboring 1, 2, 3 or more parasites at 24, 48 and 72 hours p.i. The results are shown as mean ± SD of 3 independent experiments. (TIF) [file pntd.0003194.s001.tif]

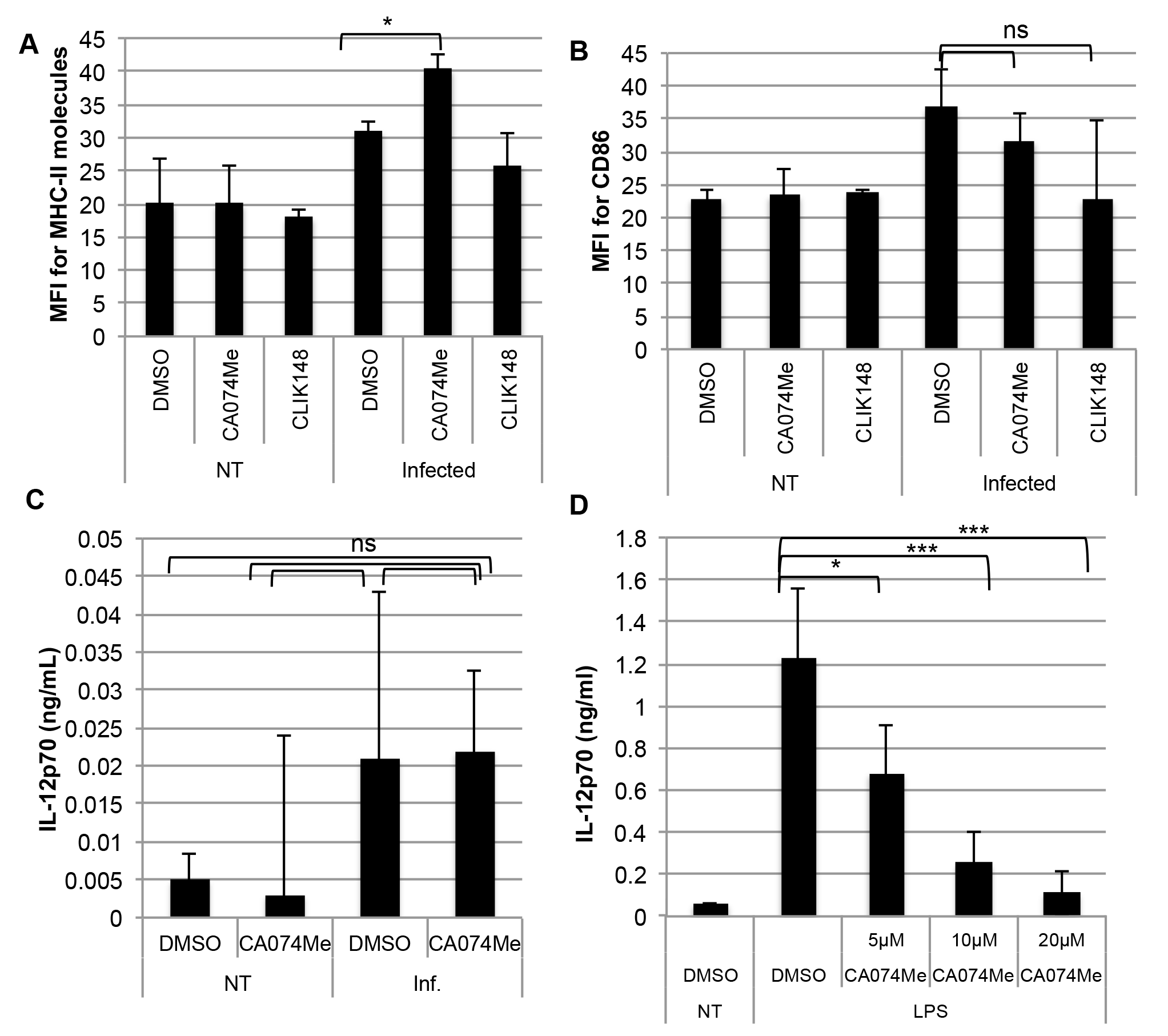

Supplement: Figure S2 — Effect of CA074Me and CLIK148 on BMDC in response to L. major promastigotes, and to LPS. BMDC were pre-incubated with the cathepsin B inhibitor CA074Me (10 µM), the cathepsin L inhibitor CLIK148 (10 µM), or an equivalent volume of DMSO for 4 hours, followed by infection with L. major promastigotes. The levels of MHC class II molecules (A) and CD86 (B) were determined by flow cytometry. (C) Concentration of IL-12p70 in the supernatants of BMDC pre-incubated with CA074Me and infected with L. major promastigotes. (D) Measurement of IL-12p70 in supernatants of BMDC stimulated with LPS in the presence of different concentrations of CA074Me. The results are shown as mean ± SD of 3 independent experiments. The statistical significance in infected cells in (A), (B) and (C) was estimated between BMDC pre-incubated with DMSO and CA074Me, and between BMDC pre-incubated with DMSO and CLIK148, * p<0.05. The statistical significance in (D) was calculated for each CA074Me concentration against LPS-stimulated BMDC pre-incubated with DMSO. * p<0.05, *** p<0.005. (TIF) [file pntd.0003194.s002.tif]

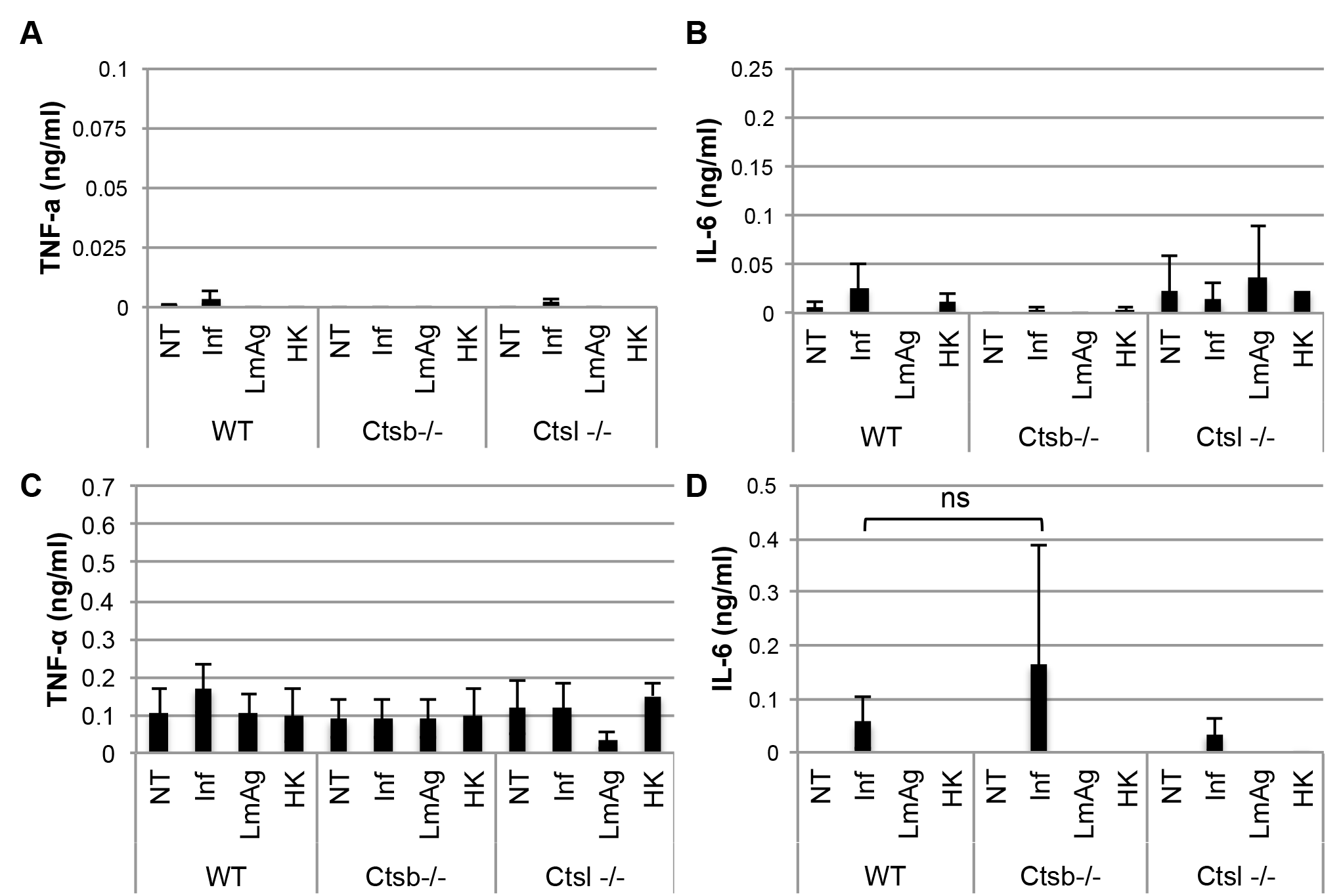

Supplement: Figure S3 — BMDC and BMM from WT and cathepsin-deficient mice express similar levels of IL-6 and TNF-α in response to L. major and LmAg. (A) TNF-α in supernatants from non-treated BMDC (NT), BMDC infected (Inf) with L. major promastigotes at 48 hours p.i. and BMDC stimulated with parasite lysate (LmAg) or heat-killed parasites (HK) for 48 hours. (B) IL-6 concentration in supernatants of BMDC at 48 hours p.i. (C) TNF-α in supernatants from non-treated BMM (NT), BMM infected (Inf) with L. major promastigotes at 48 hours p.i. and BMM stimulated with LmAg or HK for 48 hours. (D) IL-6 concentration in supernatants of BMM at 48 hours p.i. The results are expressed as mean ± SD of 3 independent experiments. For each treatment (NT, Inf, LmAg, and HK), statistical significance was assessed between WT and Ctsb−/− cells, and between WT and Ctsl−/− cells, and in all cases no statistical significance was found (p>0.05). (TIF) [file pntd.0003194.s003.tif]

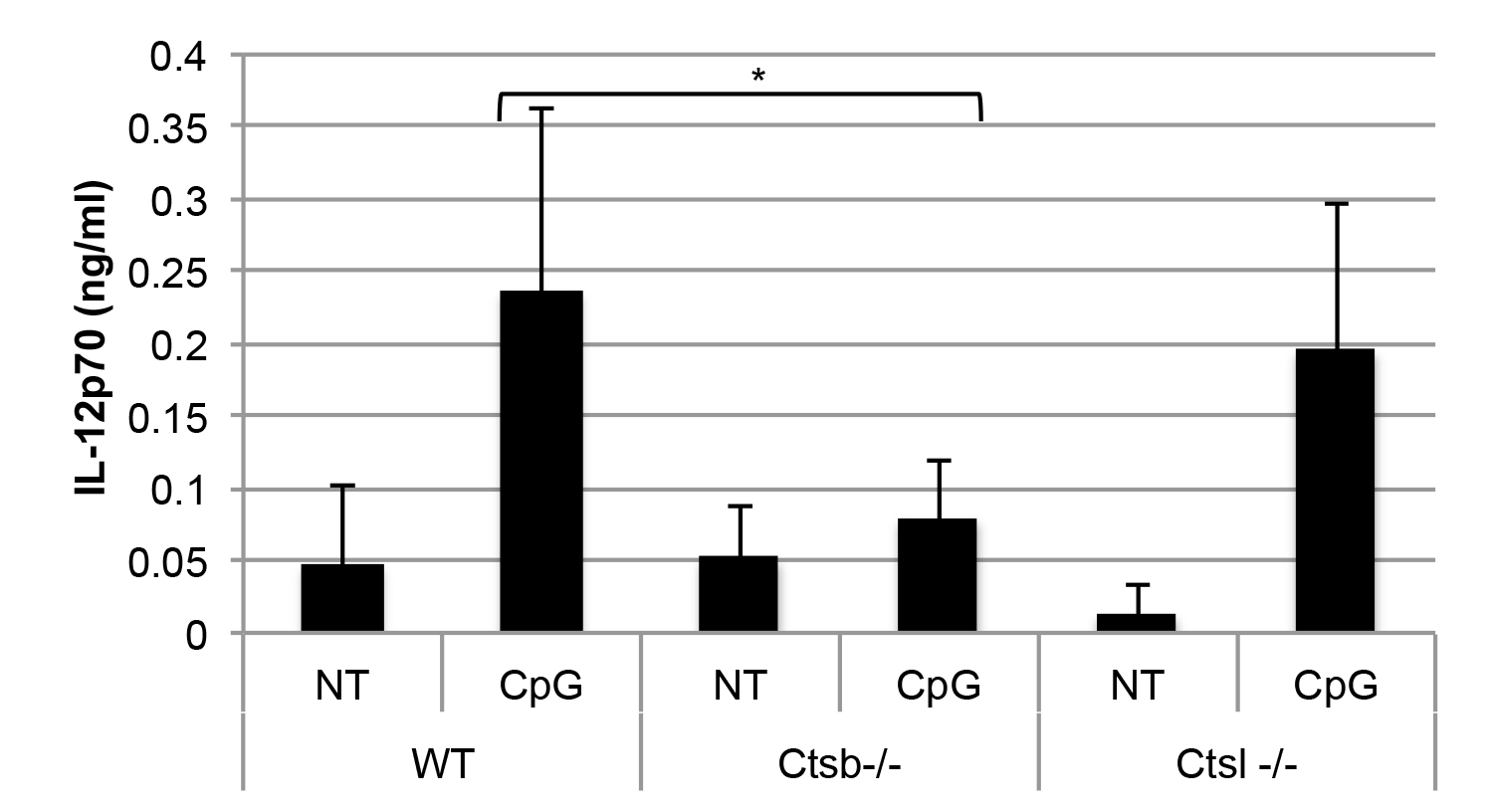

Supplement: Figure S4 — IL-12p70 expression in response to CpG is impaired in BMDC from cathepsin B-deficient mice. IL-12p70 was measured by ELISA in supernatants of non-treated (NT) or CpG-treated cells (25 µg/ml CpG, 24 hours stimulation). For each treatment, the statistical significance was calculated between WT and Ctsb−/− BMDC, and WT and Ctsl−/− BMDC. *p<0.05, ***p<0.005. (TIF) [file pntd.0003194.s004.tif]

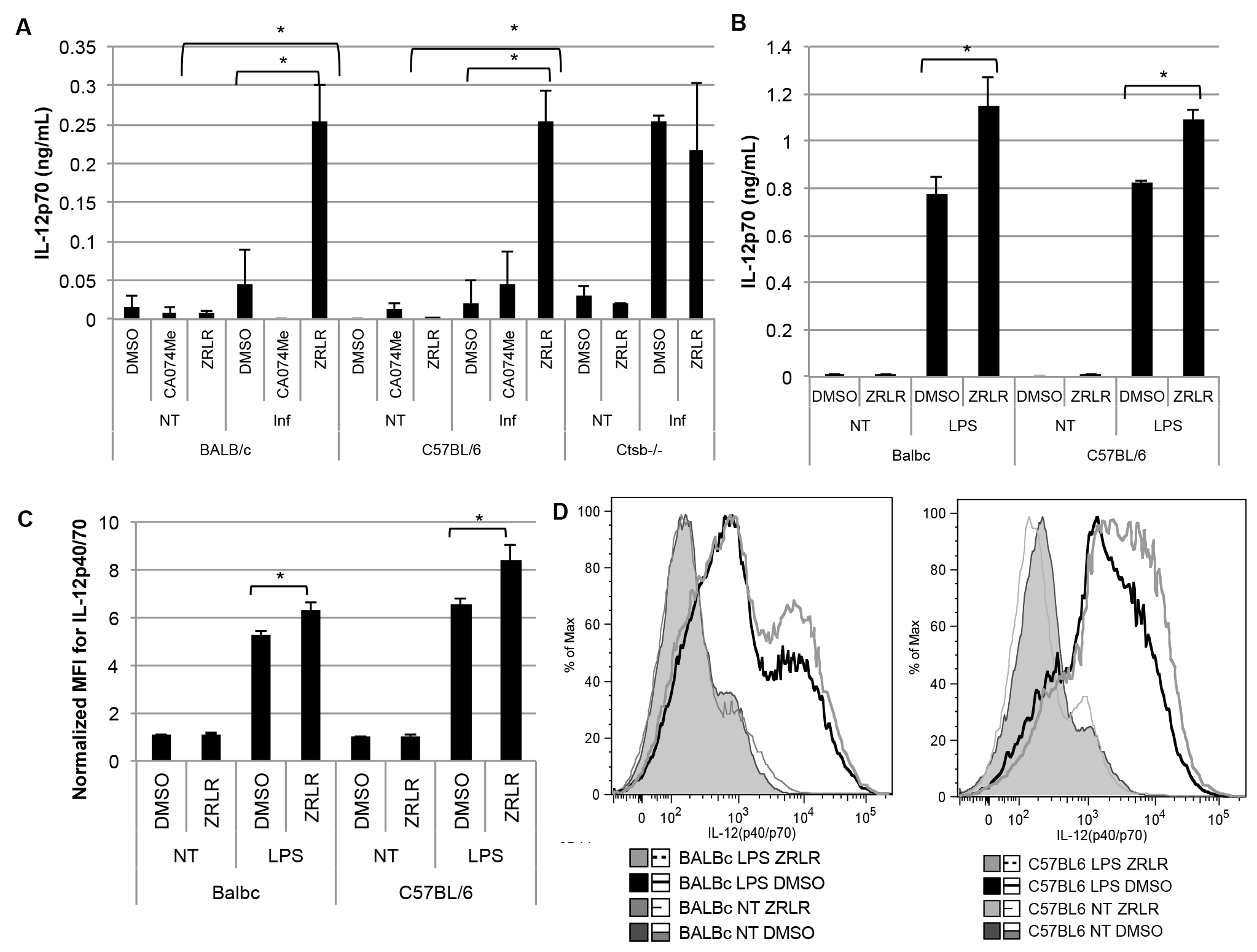

Supplement: Figure S5 — Expression of IL-12 in BMDC of BALB/c and C57BL/6 mice in response to different stimuli after inhibition of cathepsin B with ZRLR. (A) Measurement of IL-12p70 in supernatants from BMDC pre-incubated with 10 µM ZRLR, 10 µM CA074Me or DMSO, washed, and subsequently exposed to L. major promastigotes for 48 h. (B) Measurement of IL-12p70 by ELISA in supernatants of BMDC from BALB/c and C57BL/6 mice after 24 hours of stimulation with LPS in the presence of ZRLR or DMSO. The bars represent the average results from 3 independent experiments ± SD. IL-12(p40/p70) additionally was measured by intracellular staining. (C) MFI for IL-12(p40/p70); the bars represent the average MFI values from 3 independent experiments, normalized to the MFI values of NT DMSO C57BL/6 ± SD. (D) IL-12(p40/p70) histograms from one representative experiment. (TIF) [file pntd.0003194.s005.tif]

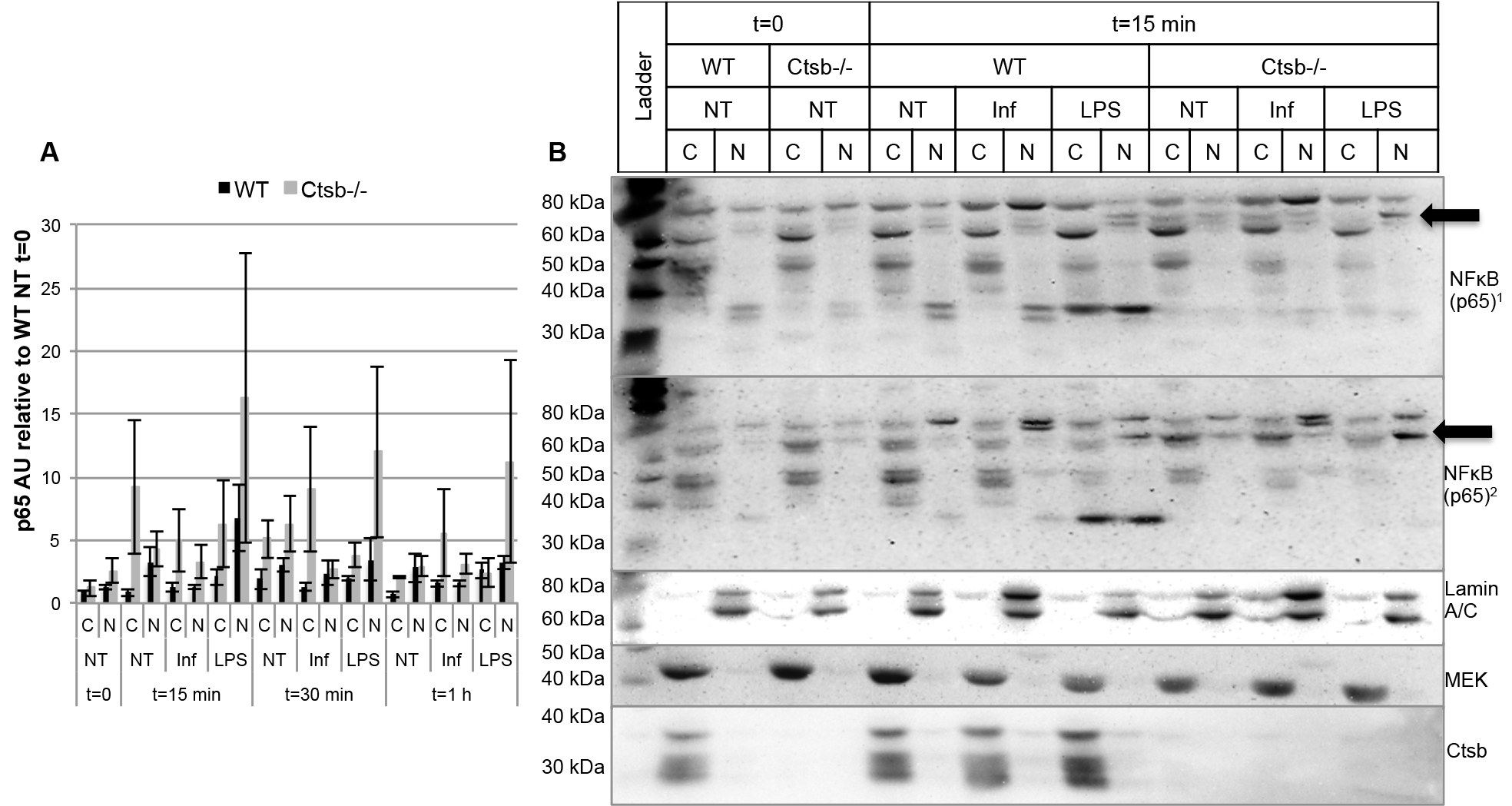

Supplement: Figure S6 — Measurement of NFκB (p65 subunit) in nuclear and cytoplasmic extracts by western blot. Nuclear (N) and cytoplasmic (C) extracts were prepared from WT and Ctsb−/− BMM at different time points after infection with L. major promastigotes or stimulation with LPS. (A) Quantification of NFκB (p65 subunit) by Western Blot, represented as arbitrary units (AU) relative to the measurements in WT BMM NT at t = 0 min. The bars represent the average result from 3 independent experiments ± SD. For each treatment, no statistical significance was found between samples from WT and Ctsb−/− BMM. B) Representative immunoblots from one experiment including samples at t = 0 and t = 15 min. Multiple bands were detected independently using two different antibodies against NFκB (p65 subunit) 1: from Santa Cruz, 2: from Cell Signaling, however only those with an apparent molecular weight of 65 kDa (black arrows) were considered for the analysis in (A). The expression levels of MEK and Lamin A/C were used as loading controls for cytoplasmic and nuclear extracts, respectively. (TIF) [file pntd.0003194.s006.tif]

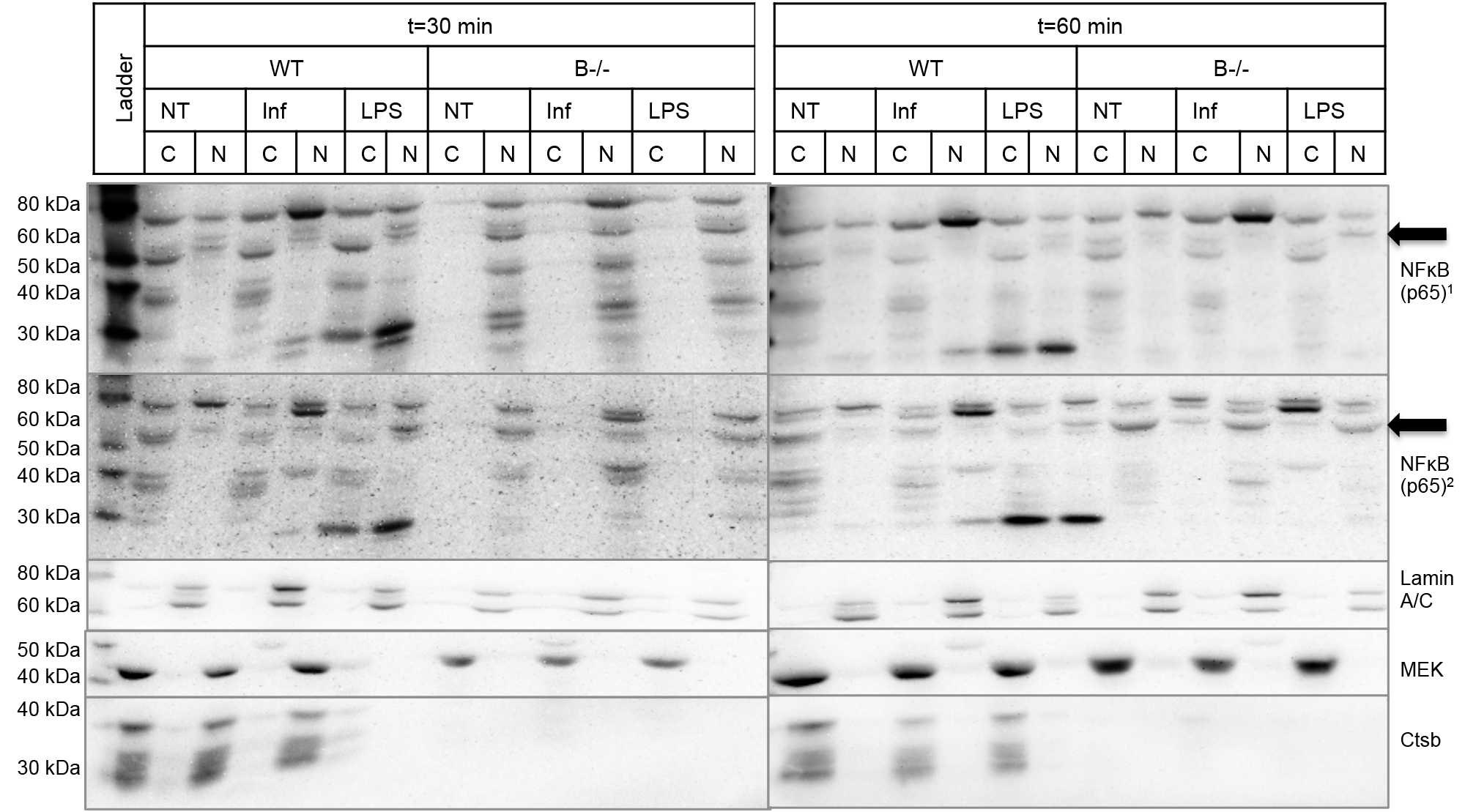

Supplement: Figure S7 — Measurement of NFκB (p65 subunit) in nuclear and cytoplasmic extracts by western blot (continuation of Figure S6). Representative immunoblots from one experiment, same as shown in Figure S6 B, including samples at t = 30 min and t = 60 min. (TIF) [file pntd.0003194.s007.tif]
